# From genomics to domestication: biocultural history of the Neotropical palms *Acrocomia aculeata* and *Acrocomia totai*

**DOI:** 10.1093/aob/mcaf282

**Published:** 2025-11-17

**Authors:** Eduardo Antonio Monge-Castro, Jonathan Morales-Marroquín, Brenda Gabriela Díaz-Hernández, Suelen Alves Vianna, Ana Flávia Francisconi, Caroline Bertocco Garcia, Matheus Scaketti, Flaviane Malaquias Costa, Alessandro Alves-Pereira, Carlos Augusto Colombo, Maria Imaculada Zucchi

**Affiliations:** Department of Genetics, Luiz de Queiroz College of Agriculture, University of São Paulo, São Paulo 13418-900, Brazil; Department of Genetics, Luiz de Queiroz College of Agriculture, University of São Paulo, São Paulo 13418-900, Brazil; Centro de Estudios Ambientales y Biodiversidad, Universidad del Valle de Guatemala, Guatemala 01015, Guatemala; Center of Plant Genetic Resources, Agronomic Institute (IAC), Campinas, São Paulo 13020-902, Brazil; Department of Research & Innovation, Plant Breeding Division, Acelen Renewable Energy, São Paulo 04794-000, Brazil; Department of Genetics, Luiz de Queiroz College of Agriculture, University of São Paulo, São Paulo 13418-900, Brazil; Department of Genetics, Luiz de Queiroz College of Agriculture, University of São Paulo, São Paulo 13418-900, Brazil; Department of Genetics and Molecular Biology, Institute of Biology, State University of Campinas, São Paulo 13083-894, Brazil; Department of Genetics, Luiz de Queiroz College of Agriculture, University of São Paulo, São Paulo 13418-900, Brazil; Department of Genetics, Luiz de Queiroz College of Agriculture, University of São Paulo, São Paulo 13418-900, Brazil; Center of Plant Genetic Resources, Agronomic Institute (IAC), Campinas, São Paulo 13020-902, Brazil; Department of Genetics, Luiz de Queiroz College of Agriculture, University of São Paulo, São Paulo 13418-900, Brazil; Department of Genetics and Molecular Biology, Institute of Biology, State University of Campinas, São Paulo 13083-894, Brazil; Paulista Agency of Agribusiness Technology, Centro-Sul Site (APTA), Piracicaba, São Paulo 13413-030, Brazil

**Keywords:** Aceraceae, *Acrocomia aculeata*, *Acrocomia totai*, ddGBS, SNP, ecological niche modelling, population genomics, archaeobotany, just energy transition

## Abstract

**Background and Aims:**

*Acrocomia* is a Neotropical palm genus that recently gained attention for its potential as a multipurpose crop. Among its species, *A. aculeata* and *A. totai* stand out for their potential in sustainable biofuel production and ecosystem restoration. Despite their relevance, the genomic structure and domestication history of these species remain poorly understood. This study aims to characterize the genetic structure and diversity of *A. aculeata* and *A. totai* across their natural distribution, to understand the biogeographical processes behind their differentiation, and to investigate the domestication history of *A. aculeata* through a genetic, ecological, archaeobotanical and ethnographic lens.

**Methods:**

We used double-digest genotyping-by-sequencing to analyse 85 individuals of *A. aculeata* and 11 of *A. totai* from nine countries. Genomic structure was assessed using sparse non-negative matrix factorization, discriminant analysis of principal components and neighbour-joining methods. For *A. aculeata*, we performed ecological niche modelling during the Pliocene and genome scans to identify outlier single nucleotide polymorphisms (SNPs) within the major genetic groups. These results were integrated with archaeobotanical and ethnographic data to contextualize domestication patterns.

**Key Results:**

We identified nine highly structured genetic clusters with low gene flow, confirming two major gene pools: Central and South America, shaped by Pleistocene–Holocene biogeographical dynamics. Using SNPs under selection, we found three regional clusters in *A. aculeata*: Central America, Amazonia and southeastern Brazil. Functional annotation revealed lineage-specific genes linked to agronomic traits: disease resistance, dwarfism and fruit development in Central America, and lipid metabolism and transcriptional regulation in South America, which may be related to independent domestication pathways over the past 13 000 years.

**Conclusions:**

Our findings offer new insights into the evolutionary and biocultural history of *Acrocomia*, supporting the existence of distinct evolutionary trajectories. These results highlight the species’ potential for sustainable development and emphasize the need for just strategies that include traditional communities in contemporary production systems.

## INTRODUCTION

*Acrocomia* is a Neotropical palms genus that has recently gained attention for the potential of its species as multipurpose crops (De Lima *et al*., 2018; Vargas-Carpintero *et al*., 2022). For most *Acrocomia* species, various parts of their fruit can be used in different ways due to their richness in carotenoids, oil, fibre and proteins, offering opportunities for applications in the pharmaceutical and cosmetic industries (Santos *et al*., 2025), food industry (Sant’ Ana *et al*., 2023) and oil extraction for biodiesel production (Colombo *et al*., 2018). These palms have received particular interest due to the species’ high oil content, comparable to that of *Elaeis guineensis* Jacq. (African oil palm), positioning *Acrocomia* species as a promising resource for energy transition and decarbonization efforts (Colombo *et al*., 2018; Solidario De Souza Benatti *et al*., 2025).

Among the species, two have gained particular interest due to their high productive potential. *Acrocomia aculeata* (Jacq.) Lodd. ex Mart. (popularly known as macaúba, coyol, corozo or macaw palm) has been highly valued owing to its wide geographical distribution, occurring in Mexico, Central America, Caribbean islands and parts of South America (Vianna and Campos-Rocha, 2025) and ability to thrive in transitional zones, from dense forests to degraded and arid areas, making it suitable for ecosystem restoration programmes (Colombo *et al*., 2018; De Lima *et al*., 2018). *Acrocomia totai* Mart. (commonly known as Mbocayá, bocaiuva, totai, bocaiuveira, macabira or mocajuba) occurs predominantly in non-flood-prone areas of the Pantanal region of Brazil, the Paraguayan–Bolivian Chaco, eastern Paraguay and northern Argentina (Vianna and Campos-Rocha, 2025), making it particularly well-suited for development programmes in South America. Paraguay currently leads in the development of a consolidated value chain for *A. totai* (Lapuerta *et al*., 2014), with ongoing efforts to expand such systems to other regions of Brazil (Vargas-Carpintero *et al*., 2022).

Advancing breeding and production efforts require a thorough understanding of natural populations to identify germplasm with desirable agronomic traits, as well as the connections among genotype, phenotype and environment that may influence oil development in *Acrocomia* (Couto *et al*., 2024). Initial efforts in this area have confirmed that *A. totai* is a distinct species from *A. aculeata* and have revealed two major genetic groups in the latter: one in Central America and the other in South (Díaz-Hernández *et al*., 2021; Morales-Marroquín *et al*., 2025). More recently, Morales-Marroquin *et al*. (2025) provided a detailed analysis of the Central American genetic pool. However, further studies are needed to better understand South American genetic diversity, the gene flow between these groups and the importance of admixture between *Acrocomia* species, and to explore the hypothesis of domestication of the species.

Domestication is a co-evolutionary process in which conscious or inadvertent human selection alters the genetic architecture of plants to produce phenotypes better suited to human needs (Clement, 1999). It is a multigenerational process in which plants can exhibit a continuum of domestication traits, ranging from non-domesticated, to incipiently and semi-domesticated, to fully domesticated forms (Clement, 1999; Clement *et al*., 2015). In the Neotropics, this process is fluid and closely intertwined with the varying modalities and intensities of human activities, resulting in a mosaic-like pattern of domestication mainly driven by Indigenous peoples (Piperno, 2011; Cámara-Leret *et al*., 2019; Iriarte *et al*., 2020; Clement *et al*., 2021; Allaby *et al*., 2022). This perspective is particularly important for palms, where the domestication syndrome is expressed across a broader morphological spectrum and is not as ubiquitous as in cereals and legumes (Fuller *et al*., 2023). Within this framework, we approach the case of *A. aculeata* not by aiming to classify it as strictly ‘domesticated’ or ‘non-domesticated’, but rather by examining how these plants were part of human landscape management and the influence on them over time.

Given the complexity of domestication processes, a multidisciplinary approach is essential. Archaeobotanical research provides direct evidence of plant use by pre-Columbian societies, especially for palms, whose remains are often well preserved through charring and whose phytoliths exhibit distinctive morphologies (Piperno *et al*., 2019; Morell-Hart *et al*., 2023). In the case of *A. aculeata*, such studies have revealed possible patterns of human-mediated dispersal, as well as early signs of selection for fruit size (Lentz, 1990). However, archaeobotanical research in South America remains scarce, and many areas are still underexplored (Iriarte *et al*., 2020; Peripato *et al*., 2023). For this reason, archaeological findings can be enriched through ethnobiological and linguistic studies that connect past uses with current practices, helping to reconstruct long-term domestication trajectories (Levis *et al*., 2018; Cámara-Leret *et al*., 2019; Costa *et al*., 2024).

One of the most informative lines of evidence in domestication research comes from genetic studies (Alam and Purugganan, 2024). In this context, studying current genomic population structure is important to reveal the evolutionary and ecological forces that shape the genetic variation of domesticated species (Allaby *et al*., 2022; Huang *et al*., 2022). In the case of *Acrocomia*, such knowledge can identify improvement genes to accelerate current efforts in *de novo* domestication programmes (Huang *et al*., 2022), as demonstrated by recent studies (Couto *et al*., 2024; Morales-Marroquín *et al*., 2025). Because domestication represents an evolutionary process, it must be analysed within the broader socio-ecological context in which the species exists (Clement *et al*., 2021; Fuller *et al*., 2023; Gepts, 2023). Therefore, ecological approaches, such as ecological niche modelling, have been increasingly used to test biogeographical hypotheses about natural and human-mediated dispersal, and to identify potential geographical or environmental barriers (Clement *et al*., 2017; Maezumi *et al*., 2018).

One of the current goals of breeders is to develop domesticated populations of *Acrocomia* for high oil yield and scalable production. Some populations of *A. aculeata* are considered to be incipiently domesticated (Clement, 1999), although some authors have suggested that they may be semi-domesticated (Lentz, 1990; Cooke, 1992; Morcote-Ríos and Bernal, 2001). There are two main hypotheses about its domestication: one favours an origin from Mexico from which it dispersed to South America (Lentz, 1990), and the other proposes a South American origin with northward dispersal to Central America (Morcote-Ríos and Bernal, 2001). Both hypotheses are based on archaeobotanical records and ecological evidence, suggesting that *A. aculeata* is a pioneer species that thrives in human-disturbed areas which may have facilitated its spread across landscapes modified by early human populations (Ford *et al*., 2023). To date, however, no studies have explicitly investigated these hypotheses or the broader domestication process in depth, neither for *A. aculeata* nor for *A. totai.*

Understanding domestication not only reveals the evolutionary trajectories of crops, but may also contribute to more sustainable and just agricultural management practices (Clement *et al*., 2021; Gepts, 2023). As breeding programmes for *A. aculeata* begin to take shape, unravelling its diversification and domestication history is essential, not only to guide genetic improvement but also to recognize the key communities that have shaped and maintained this species across the Neotropics (Cámara-Leret *et al*., 2019; Vargas-Carpintero *et al*., 2021). In this context, this study aimed to: (1) characterize the genetic structure of populations throughout the species’ distribution and to discuss the biogeographical processes that have shaped it; and (2) to examine how the observed genetic signatures may have been influenced by ecological, archaeobotanical and ethnographic data to better understand the domestication process of *A. aculeata*. This knowledge will be valuable to support ongoing domestication efforts while emphasizing the need to include historically connected Latin American communities in shaping the future of this native resource.

## MATERIALS AND METHODS

### Plant material and DNA isolation

**Fig. 1.**
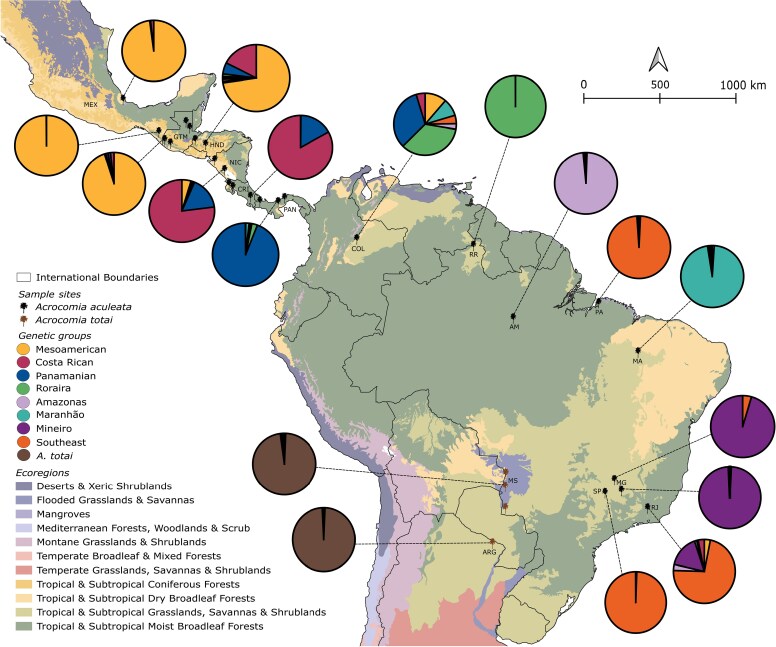
Sampling locations and genetic structure suggested by sparse non-negative matrix factorization (sNMF) based on 4716 SNPs from 70 samples of *Acrocomia aculeata* and 11 of *A. totai*. Pie charts show the average sNMF ancestry coefficients for each genetic group represented by different colours. The different ecoregions are defined according to Dinerstein *et al*. (2017). Acronyms for countries and states: MEX, Mexico; GTM, Guatemala; HND, Honduras; NIC, Nicaragua; CRI, Costa Rica; PAN, Panama; COL, Colombia; Brazilian States: MA, Maranhão; PA, Pará; AM, Amazonas; RR, Roraima; MG, Minas Gerais; SP, São Paulo; and RJ, Rio de Janeiro.

For this study, samples obtained from Díaz-Hernández *et al*. (2021) and Morales-Marroquin *et al*. (2025) were selected to represent the natural distribution of *A. aculeata* and *A. totai*, as well as the different genetic groups previously described. We selected 96 samples: 11 for *A. totai* and 85 for *A. aculeata* collected from Mexico, Guatemala, Honduras, Costa Rica, Nicaragua, Panama, Colombia, Brazil and Argentina (Fig. 1; Supplementary Data Table S1). DNA was extracted from leaf tissues using the Doyle and Doyle (1990) protocol and stored at −80 °C. We assessed the quality and quantity with electrophoresis in 1 % agarose gels stained with GelRed®, and Qubit broad-range DNA assay kit (Qubit – Life Technologies). DNA concentrations were normalized to 30 ng μL^−1^.

### Library preparation and SNP calling

We prepared a double-digest genotyping-by-sequencing (ddGBS) genomic library following the protocol devolved by Poland *et al*. (2012). We digested the DNA using the combination of MseI and PstI (New England Biolabs Inc.) enzymes. The digested fragments were ligated to MseI adapters (containing barcode sequences) and PstI adapters (containing Illumina^TM^ adapters). The library was enriched through polymerase chain reaction (PCR) using Phusion High-Fidelity PCR Master Mix (New England Biolabs Inc.). After PCR the library was purified using AMpure® XP magnetic beads (Agencourt®). The library was sequenced using a NextSeq 1000/2000 P2 flow cell in an Illumina NextSeq2000 platform (Illumina Inc.) with single-end configuration for 100 bp.

We assessed the quality of the sequenced library using FastQC. Trimming the reads to 90 bp and demultiplexing were performed using the *process_radtags* module of Stacks 2.68 (Rochette *et al*., 2019). We aligned the reads to the *A. aculeata* genome assembly (Scaketti *et al*., 2025) using the bwa-mem algorithm from BWA 0.7.1 (Li 2013). The aligned files were manipulated with Picard 4.1.3.0 (Picard, 2019), and the genetic variants were identified using FreeBayes 0.9.21 (Garrison and Marth, 2012) with the *standard_filters* option. Single nucleotide polymorphism (SNP) loci were filtered using VCFtools 0.1.16 (Danecek *et al*., 2011) and BCFtools 0.1.12 (Danecek *et al*., 2021) to retain only biallelic SNPs with a minor allele frequency (MAF) ≥ 0.01, sequencing depth ≥ 5×, mapping quality ≥ 20, a maximum of 10 % missing data per locus, minimum distance of 150 bp between loci and *r*^2^ < 0.6 to avoid explicit linkage disequilibrium (LD) (Supplementary Data Fig. S13). A final filtering step was performed to exclude samples with more than 25 % missing data.

### Population genomics analyses

To assess the population structure we used complementary analyses: a sparse non-negative matrix factorization (sNMF) (Frichot *et al*., 2014), discriminant analysis of principal components (DAPC) (Jombart *et al*., 2010), and the neighbour-joining (NJ) method (Saitou and Nei, 1987) based on Nei’s (1987) genetic distance. For sNMF we tested from 1 to 10 *K* ancestral populations with 10 repetitions and 10 000 000 iterations for each *K* value to ensure convergence, using the cross-entropy criterion to choose the most likely number of clusters. We performed this analysis using LEA 3.16.0 (Frichot and François, 2015; Gain and François, 2021). For DAPC, we used the function *find.clusters* to identify the number of clusters using k-means algorithms, choosing the number of clusters based on the Bayesian information criterion (BIC) (Supplementary Data Fig. S1A). Then we used the *optim.a.score* function to retain 12 principal components (Fig. S1B) to reduce over-fitting of DAPC’s membership coefficients (Jombart *et al*., 2010). The analysis was performed in adegenet 2.0.10 (Jombart and Ahmed, 2011). The NJ tree based on Nei’s (1987) genetic distance was built using the *aboot* function from poppr 2.9.6 (Kamvar *et al*., 2014) with 1000 bootstrap replicates. The final tree was formatted using TreeViewer 2.2.0 (Bianchini and Sánchez-Baracaldo, 2024).

We determined the number of populations based on the concordance among these different methods. We calculated for each population the total number of alleles (*A*) and number of private alleles (*A*_P_) using adegenet 2.1.10 (Jombart and Ahmed, 2011) and poppr 2.9.6 (Kamvar *et al*., 2014). Inbreeding coefficients (*f*), allelic richness (*A*_R_), observed heterozygosities (*H*_O_) and gene diversity (*H*_S_) were estimated using hierfstat 0.5-11 (Goudet, 2005). Genetic divergence among populations was assessed through pairwise Wright’s *F*_ST_ (Wright, 1949) based on Nei’s distance (Nei, 1987), and results were visualized with heatmaps generated by pheatmap 1.0.12 (Kolde, 2018). Indirect estimates of gene flow among populations were obtained following the Slatkin (1987) formula: *Nm* = (1 − *F*_ST_)/4*F*_ST_, with the igraph 2.1.4 package (Csardi and Nepusz, 2006). While we recognize that this model relies on simplifying assumptions (Whitlock and McCauley, 1999), our goal is not to estimate absolute migration rates, but to visualize broad trends in genetic exchange among populations, supporting broader interpretations of the species’ evolutionary history. Accordingly, the graph was constructed using only neutral SNPs for *A. aculeata*.

### Domestication processes of *Acrocomia aculeata*

#### Detection of SNPs under putative selection

To deepen our understanding of the historical use and potential domestication processes of *A. aculeata* through an integrative analysis of genetic, ecological and archaeological evidence, we performed complementary analyses using only outlier SNPs, which are putatively under selection. These SNPs were chosen because of their potential to reflect selective pressures (Morales-Marroquín *et al*., 2025; Pantoja *et al*., 2025) such as those possibly imposed by human use or adaptation to distinct environments. To explore both general patterns and region-specific processes, we generated three datasets, and outlier SNPs were identified for each of them. The first included individuals from both Central and South American groups (*n* = 70), the second included only Central American individuals (*n* = 35) and the third included only South American individuals (*n* = 35).

Outlier SNPs were identified using four methods [pcadapt, bayescan, fsthet and latent factor mixed model (LFMM)]. The pcadapt package (Luu *et al*., 2017) detects outlier loci that present the greatest contribution to genetic structure based on principal component analysis (PCA) without assuming any genetic model. The analysis was performed using pcadapt 4.3.5 (Privé *et al*., 2020), testing from 1 to 20 *K* principal components. SNPs were considered outliers, retaining *K* = *2* for the three databases (Supplementary Data Fig. S3) and loci with q-values ≤ 0.1.

BayeScan (Foll and Gaggiotti, 2008) employs a Bayesian framework to decompose the *F*_ST_ values into two components: a population-specific component (β) and a locus-specific component (α). The software then uses a logistic regression reversible-jump Markov chain Monte Carlo (RJ-MCMC) algorithm to determinate if the alpha component deviates significantly from a neutrality model, indicating potential local adaptation when α > 0. The analysis was run using BayeScan 2.1 (Foll and Gaggiotti, 2008) with 20 pilot runs of 5000 iterations, a burn-in of 50 000 iterations, a thinning interval of 10 and thinning interval size of 10. Outlier loci were identified as those with *q*-values ≤ 0.1.

The fsthet package (Flanagan and Jones, 2017) identifies outlier SNPs based on the *F*_ST_–heterozygosity relationship, detecting loci with *F*_ST_ values significantly above or below the expected range under neutrality. Smoothed quantiles of the *F*_ST_–heterozygosity distribution were calculated using 1000 bootstraps, with loci falling outside the 95 % confidence intervals considered outliers. This analysis was conducted using fsthet 1.0.1 (Flanagan and Jones, 2017).

We used LFMM (Frichot *et al*., 2013) to correlate SNP markers with the WorldClim2 variables (Fick and Hijmans, 2017), estimating latent factors based on an exact least-squares approach. First we obtained the elevation and the 19 WorldClim2 environmental variables at 2.5-min resolution (Hijmans *et al*., 2024). To determinate the environmental variables to use in the model, we conducted a PCA and retained PCs that explained at least 90 % of the observed variance. The variables retained for the model were BIO6, BIO12, BIO15 and BIO7 for the complete dataset; for the Central American group, BIO9, BIO16, BIO7 and BIO4; and for the South American group, BIO6, BIO13 and BIO15 (Supplementary Data Fig. S4; Table S2). Then, an sNMF analysis (Frichot *et al*., 2014) was performed to estimate the most likely number of genetic clusters for each dataset, following the same parameters as before. Based on the cross-entropy criterion, *K* = 8 was most probable for the complete dataset, *K* = 3 for Central America and *K* = 5 for South America (Fig. S7). These results were used to perform the LFMM analyses with 10 repetitions of 50 000 burn-in followed by 100 000 iterations. Both sNMF and LFMM were run with LEA 3.16.0 (Frichot and François, 2015; Gain and François, 2021). We consider as outliers those SNPs that presented *P*-values less than the false discovery rate (FDR) of 0.1.

We considered SNPs putative under selection as those loci that were identified in at least two of the four methods, while the remaining loci were considered as neutral. To assess the predicted functions of outlier SNPs, we used BEDtools 2.18 (Quinlan and Hall, 2010) to recover the sequences of genes with outlier SNPs. Then, gene sequences were used as a query for local BLASTx against the *Elaeis guineensis* protein database [GCA_000442705.2] using the following configurations: *evalue* 1e-5, *-max_target* 5, *-outfmt* 6. The annotated genes were compared with the list of genes from genome-wide association studies (GWAS) available for palms, as well as with known domestication-related genes (Alam and Purugganan, 2024), to identify genes that may be associated with the historical uses of the species. To test the hypothesis of independent selection events between regions, we calculated the statistical significance of the observed intersections between the outlier sets using the SuperExactTest 1.1.0 293 package (Wang *et al*., 2015), which provides an exact test for multi-set overlaps.

#### Structure under selection

We used the set of outlier SNPs to assess the genetic structure using the same methods described above for the whole set of loci. Additionally, we conducted a spatial analysis of principal components (sPCA) (Jombart *et al*., 2008) that summarizes genetic variability while accounting for spatial correlation among populations based on a connection network. Our network was constructed using the *K*-nearest neighbour method with *K* = 20, a choice that ensured meaningful connections at a regional scale without overrepresenting local structure (Supplementary Data Fig. S14). The significance of the global and local structures was evaluated with 1000 permutations (Montano and Jombart, 2017). Spatial patterns were visualized using the functions from adegenet 2.0.10 (Jombart and Ahmed, 2011), and spatial interpolation was performed with the akima 0.6-3.4 package (Akima *et al*., 2022). To complement this, we performed a Mantel test (Mantel, 1967) to evaluate the correlation between genetic differentiation (pairwise *F*_ST_) and geographical distance (km), calculated from sampling coordinates using the geosphere package v.1.5-20 (Hijmans, 2024). The Mantel test was carried out with 10 000 permutations using the ade4 package v.1.7-23 (Dray and Dufour, 2007).

#### Archaeobotanical remains

We projected the genetic groups under selection identified by the sNMF onto a map of the main linguistic families present in America at 1500 CE (adapted with authorization from Bellwood, 2024). We performed this association to study possible patterns related to use by Indigenous societies, assuming that by the time of the European conquest, there was still a relationship between the geographical location of the samples and the native languages spoken in those areas. We also plotted in this map the locations and ages of archaeological records. This information was obtained from a literature review, which was conducted in Spanish, English and Portuguese, utilizing the common names in the three languages (bocoyá, totaí, korondía, macaúba, mucajá, côco de catarro, maka-djiup, corozo, tamaco, coqueiro de catarro, Mbocayá, coyol, cocoyol and macaw palm; Dransfield *et al*., 2008; Smith, 2015; De Lima *et al*., 2018), the current scientific name [*Acrocomia aculeata* (Jacq.) Lodd. ex Mart.; Vianna and Campos-Rocha, 2025) and the accepted synonyms for the species (*Cocos aculeata*, *A. globosa*, *A. mexicana*; Dransfield *et al*., 2008; Vianna and Campos-Rocha, 2025). Only information from scientific articles, books or academic theses that provided a specific date for the record, a specific archaeological site and the type of material used (e.g. cords, phytoliths or macro-remains) was included. When available we also record the context of the palm’s use (food/cooking, ceremonial/religious or construction) (Supplementary Data Table S5). With this, we wanted to speculate how the patterns of genetic structure based on outlier SNPs were possibly related to use by American societies that were using *A. aculeata* at the time of European conquest.

#### Ecological niche modelling

We conducted an ecological niche modelling analysis during the Pleistocene to assess how climatic events influenced the species’ distribution, considering both its distribution prior to human intervention, as well as its availability for human populations during this time. We used the Global Biodiversity Information Facility (GBIF) accessed on 24 February 2025 as the primary source of information of occurrence (GBIF.org, 2025). To complement this we use the data available from the UICN Red List (Machuca Machuca *et al*., 2022), Flora Mesoamericana (Tropicos v.3.4.2) on 24 February 2025 (Missouri Botanical Garden, 2025), and the geo-referenced samples reported in Díaz-Hernández *et al*. (2021) and Morales-Marroquin *et al*. (2025). The final dataset selected for modelling included 4417 occurrences from 14 countries (Supplementary Data Table S3). For the past bioclimatic variables we used the PaleoClim dataset (Fordham *et al*., 2017; Brown *et al*., 2018) with 2.5 arc-min (∼5 km) of resolution. We used four different dates: Last Interglacial (ca. 130 ka), Last Glacial Maximum (ca. 21 ka), Younger Dryas Stadial (12.9–11.7 ka) and Northgrippian (8.326–4.2 ka). As a base-map we use the Anthropocene (1979–2013) data from CHELSA (Karger *et al*., 2017).

To perform the modelling, we used the workflow implemented in ENMTML 1.0.0 (Andrade *et al*., 2020). To reduce autocorrelation in the data, we applied a method based on cell size, which selects pairs of occurrences within a 2× cell-size distance (Velazco *et al*., 2019). To control for collinearity in predictors, we used the PCA method, which performs PCA on standardized variables and uses PCs as new variables (De Marco and Nóbrega, 2018). Pseudo-absences were allocated using the environmental constraint method, based on the lowest suitable region predicted by a Bioclim model (Engler *et al*., 2004; Booth *et al*., 2014). To partition the occurrence data, we applied the bootstrap method with 70 % for training and 30 % for validation (Fielding and Bell, 1997). The final models were constructed using four different algorithms: Maximum Entropy (Maxent) (Phillips and Dudík, 2008), Random Forest (Liaw and Wiener, 2002), Bioclim (Booth *et al*., 2014) and Support Vector Machine (Guo *et al*., 2005). We assembled them based on the average suitability predicted (Thuiller *et al*., 2009) using the true skill statistics (TSS) as threshold for assembly. The quality of the models was evaluated using the TSS (Allouche *et al*., 2006) and area under the curve (AUC) (Metz, 1986) metrics.

## RESULTS

### SNP genotyping

The ddGBS library resulted in 319 524 013 raw reads. After demultiplexing, trimming and filtering, 99 632 225 reads (mean = 1 610 284 reads per sample, s.d. ± 1 313 815) were used for SNP identification. Before LD pruning, we obtained a set of 10 955 SNPs, which was reduced to a final set of 4716 high-quality, LD-pruned SNPs (20.89 mean sequence depth per locus, ± 15.44 s.d., 2.36 % of missing data) present in 70 individuals of *A. aculeata* and 11 individuals of *A. totai* (Supplementary Data Table S1).

#### Genetic diversity and population structure of *Acrocomia* species

The three population structure analyses (sNMF, DAPC and NJ) indicated a high level of genetic differentiation among *Acrocomia* populations. sNMF suggested the formation of eight genetic groups for *A. aculeata* (Fig. 1; Supplementary Data Fig. S7), while DAPC suggested the formation of seven (Fig. S1). The main difference among these analyses lies in the classification of the Maranhão and Amazonas populations. While sNMF suggested they represent two distinct genomic groups, DAPC grouped them into a single cluster. The NJ tree also supported the division of Maranhão and Amazonas as two different related groups (Fig. S2).

For the rest of the populations, the three methods indicate similar results. Roraima individuals are grouped primarily with Colombia, although sNMF suggested that the Colombian population was also admixed with the Panamá samples (Fig. 1). Interestingly, individuals from Pará (northern Brazil) consistently cluster with individuals from São Paulo and Rio de Janeiro (southeastern Brazil). Individuals from Minas Gerais, despite being from southeastern Brazil, formed a distinct group. *Acrocomia totai* formed a consistent group, with some degree of admixture with individuals from the southeast.

Based on the concordance between the methods, we propose that our *Acrocomia* samples are structured in nine genetic groups (Fig. 1). For the Central American populations, we adopt the names proposed by Morales-Marroquin *et al*. (2025). The Mesoamerican group includes samples from Mexico, Guatemala and Honduras. The Costa Rican group represents a transitional cluster comprising populations from Nicaragua and Costa Rica. The Panamanian group is composed primarily of populations from Panama, with some individuals from Nicaragua and Costa Rica. For South America, we propose five genomic groups. The Roraima group includes samples from Roraima and Colombia. The population from Amazonas and the population from Maranhão formed two distinct groups. The population from Minas Gerais formed the Mineiro group. The Southeast group included samples from Pará, São Paulo and Rio de Janeiro. The last groups consist of *A. totai* samples.

**Fig. 2.**
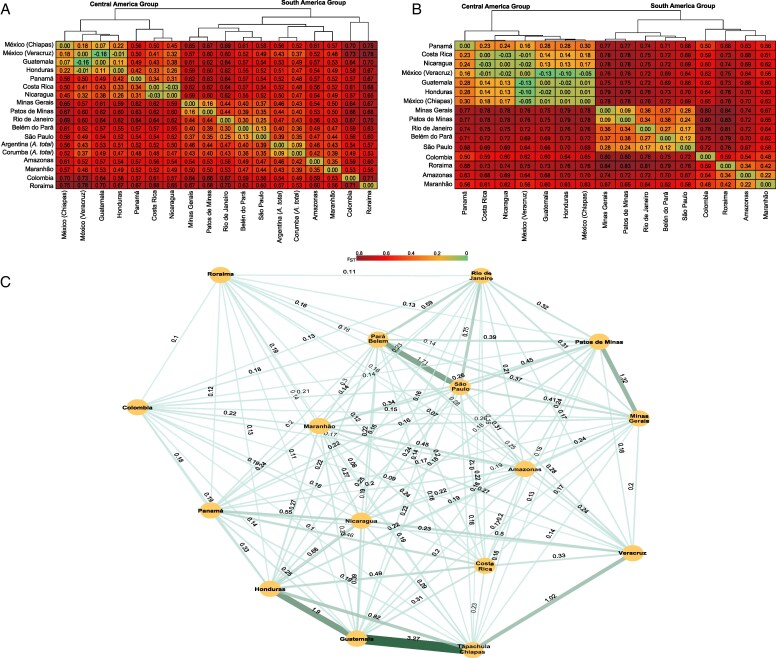
Dendrogram, heatmap and gene flow network (*Nm*) based on pairwise *F*_ST_ estimates among sampling locations. (A) *F*_ST_ estimated using 4716 SNPs from 70 samples of *A. aculeata* and 11 of *A. totai*. It also shows the division of the population into two main clusters: Central America and South America. (B) *F*_ST_ estimated using 164 outlier SNPs identified for the 70 samples of *A. aculeata*. (C) Gene flow network (*Nm*) estimated using the formula of Slatkin (1987) based on 4552 neutral SNPs from 70 samples of *A. aculeata*. Edge thickness is proportional to estimated gene flow, representing relative connectivity among populations.

Pairwise *F*_ST_ estimates supported the high structuring and differentiation of the populations, and the division into two major groups: the Central American group and the South American group (Fig. 2A). When comparing those two major groups, the *F*_ST_ values indicate very high differentiation (*F*_ST_ > 0.6). For Central America, Panamá showed higher differentiation (*F*_ST_ ranging from 0.31 to 0.56) in relation to other locations, while the lowest differentiation was observed within the Mesoamerican group (*F*_ST_ ranging from −0.16 to 0.18). For South America, in general, higher genetic differentiation was observed between groups than within groups. For example, within the Southern groups the *F*_ST_ values indicate moderate differentiation (*F*_ST_ ranging from 0.13 to 0.3), but when compared with the Mineiro group, it shows a very strong differentiation (*F*_ST_  *>* 0.4). Notably, Roraima was highly differentiated from other populations, even more than *A. totai*, with is accepted as a different species.

The gene flow (*Nm*) network (Fig. 2C) is consistent with the patterns observed in the genetic structure and *F*_ST_ analyses. It highlights Roraima samples as clearly isolated, showing limited genetic exchange with the remaining populations. In South America, the graph reinforces the distinctiveness of the Mineiro cluster, as well as gene flow between Belém and São Paulo, supporting their grouping within the Southeastern cluster. In Central America, the network illustrates notable genetic exchange within the Mesoamerican group and also suggests historical gene flow between Colombia and Panama.

In general, the South America populations had higher genetic diversity than Central American populations (Table 1). Within Central America, the Mesoamerican group presented slightly higher genetic diversity and much higher number of private alleles than the Costa Rican and Panamanian group. Within South America, the Southeast group had the highest genetic diversity while Roraima had the lowest. The Mineiro and Amazonas groups had higher number of private alleles than the other groups, reinforcing their genetic distinctiveness.

**Table 1.** Genetic diversity for nine *Acrocomia* genetic groups.

Group	*n*	*A*_R_	*A*	*A*_P_	*H*_S_ (95 %CI)	*H*_O_ (95 % CI)	*f* (95 % CI)
**Central America Major Group**							
Mesoamerican	16	1.053	5912	821	0.053 (0.050–0.057)	0.043 (0.039–0.047)	0.166 (0.155 to 0.239)
Costa Rican	11	1.051	5551	481	0.051 (0.048–0.055)	0.041 (0.037–0.045)	0.210 (0.152 to 0.246)
Panamanian	8	1.048	5563	229	0.049 (0.046–0.053)	0.041 (0.037–0.045)	0.154 (0.103 to 0.207)
**South America Major Group**							
Roraima	7	1.079	5685	189	0.081 (0.076–0.086)	0.045 (0.040–0.049)	0.388 (0.406 to 0.494)
Amazonas	5	1.096	5822	301	0.097 (0.093–0.103)	0.087 (0.082–0.093)	0.071 (0.069 to 0.136)
Maranhão	4	1.110	5848	148	0.109 (0.103–0.114)	0.115 (0.109–0.123)	−0.078 (−0.098 to −0.030)
Mineiro	7	1.108	6137	505	0.109 (0.104–0.115)	0.095 (0.089–0.100)	0.107 (0.103 to 0.162)
Southeast	12	1.138	6812	1115	0.139 (0.133–0.144)	0.118 (0.112–0.123)	0.132 (0.134 to 0.175)
*A. totai*	11	1.185	7523	3814	0.186 (0.180–0.191)	0.169 (0.163–0.175)	0.081 (0.077 to 0.111)

The estimates were obtained from 4716 SNPs for 70 samples of *A. aculeata* and 11 of *A. totai*. *n*, number of samples; *A*_R_, allelic richness; *A*, total number of alleles; *A*_P_, number of private alleles; *H*_S_, gene diversity; *H*_O_, observed heterozygosity; *f*, inbreeding coefficient; 95 % CI, 95 % confidence interval.

All groups of *A. aculeata*, except Maranhão, presented high inbreeding coefficient (*f* ranging from 0.071 to 0.388). Also, the observed heterozygosity in all cases is lower than the gene diversity, indicating potential genetic isolation or drift in the populations. This is more pronounced in the populations of Roraima, which not only have the highest inbreeding index but also exhibit an observed heterozygosity less than half of the expected value. *Acrocomia totai* had higher genetic diversity, greater number of private alleles and lower inbreeding coefficient than *A. aculeata*, indicating better maintenance of those populations.

### Domestication processes of *Acrocomia aculeata*

#### Detection of SNPs under selection

**Fig. 3.**
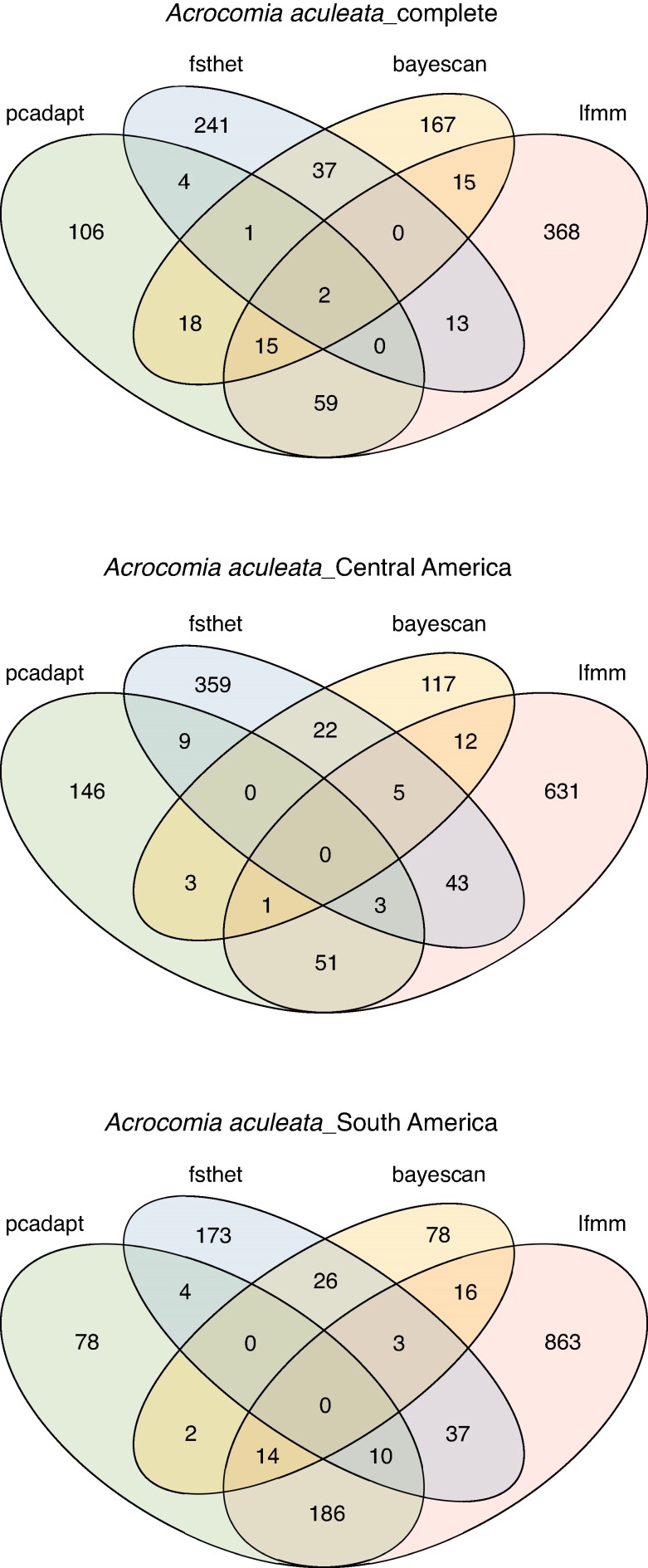
Summary of genome scans for signatures of selection considering different groups of *A. aculeata*. Venn diagrams show the number of outlier SNPs detected for each test and the overlap among them for the complete *A. aculeata* dataset (*n* = 70), the Central America major group (*n* = 35) and the South America major group (*n* = 35). We considered outlier SNPs as being present in at least two of the four methods.

Combining the four methods, 1046 outlier SNPs were detected for the complete dataset (pcadapt: 205, fsthet: 298, BayeScan: 255, LFMM: 472), 1407 for the Central America group (pcadapt: 213, fsthet:446, BayeScan:160, LFMM: 751) and 1490 for the South America group (pcadapt: 294, fsthet: 253, BayeScan: 139, LFMM: 1129) (Supplementary Data Fig. S5–S8). Considering the loci detected in at least two of the four methods, 164 outliers were identified for the complete dataset, 154 for the Central America group and 298 for the South America group (Fig. 3). Comparing the SNPs between groups, 13 SNPs are shared between South America and Central America, 13 between South America and the complete database, 73 between Central America and the complete data, and only nine are shared among the three groups (Fig. S8; Supplementary Information S2). For the complete dataset, 100 outlier SNPs were located within predicted protein-coding regions. For Central America and South America, 99 and 183 SNPs were located within coding genes, respectively.

The functional annotation of these coding regions revealed that for both groups, South America and Central America, many of the outlier SNPs were within genes involved in basic metabolic processes. Among the nine loci shared across all three datasets, most annotated were related to core functions such as cellular transport, signalling and primary metabolism (Supplementary Information S2). The Fisher/ExactTest confirmed that these 465 shared loci occur significantly more often than expected by chance (FE = 27.1, *P* < 1.466 × 10⁻^10^; Supplementary Data Table S8; Fig. S15), suggesting strong, convergent selective pressures on essential functions across regions. However, each region presented a mayor set of unique genes under selection, some of which are related to key agronomic traits (Table 2). This pattern may reflect differences in historical use and human-driven selection, suggesting independent domestication. In the Central American group, several outlier SNPs were possibly linked to traits commonly targeted in domestication, including disease resistance, fruit development and dwarf phenotypes. In contrast, for the South American group, some outlier SNPs were within genes related to lipid metabolism, fruit biomass and transcriptional regulation, aligning with selection for oil content and fruit yield. The full list of annotated genes for the three datasets is provided in Supplementary Information S2.

**Table 2.** Example of outlier SNPs within genes detected in Central and South American groups, including candidates linked to agronomic traits.

SNP ID	Outlier SNP annotation	Identity (%)	*E*-value	Associated agronomic traits	General function	Reference
**Central America Major Group**						
scaffold_2_pilon: 117763160	Zinc finger protein NUTCRACKER	90.75	2.00E-167	OC, LL-OC & LN-OC in *A. aculeata*; BW in *E. oleifera × E. guineensis*	Regulate responses to abiotic stress	(Osorio-Guarín *et al*., 2019; Couto *et al*., 2024)
scaffold_1_pilon: 117751234	Putative disease resistance protein RGA3	93.81	0	Leaf spot resistance in *E. guineensi*	Foliar disease resistance	(Wibowo *et al*., 2023)
scaffold_9_pilon: 17155492	Reduced wall acetylation 3	82.58	2.00E-92	EFM & HDM in *A. aculeata*; dwarfism phenotype in *Arabidopsis*	Cell wall polysaccharide acetylation	(Manabe *et al*., 2013)
scaffold_13_pilon: 2793710	ABC transporter B family member 20	98.78	0	Dwarf phenotypes in maize, sorghum and watermelon	Modulation of polar auxin transport	(Banasiak and Jasiński, 2022)
scaffold_10_pilo:6417576	Remorin	98.04	5.00E-23	–	Host–microbe interactions	(Lefebvre *et al*., 2010)
scaffold_9_pilon: 103726085	Glucan endo-1,3-beta-glucosidase	93.95	0	–	Protection against microbial invasion	(Costa *et al*., 2020)
**South America Major Group**						
scaffold_19_pilon: 9065646	Trihelix transcription factor GT-3b	93.72	4.00E-66	Growth–defence metabolism in maize	Plant growth	(Zhang *et al*., 2021)
scaffold_7_pilon: 5746340	Ribonuclease E/G-like	78.21	0	PFM in *A. aculeata*	Process transcripts from chloroplast operons	(Walter *et al*., 2010)
scaffold_13_pilon: 12394338	GDSL esterase/lipase	76.53	1.00E-122	FM in *A. aculeata*; OC in *E. guineensis*	Fatty acid metabolism and seed germination	(Zhang *et al*., 2018; Couto *et al*., 2024)
scaffold_13_pilon: 4240228	Omega-3 fatty acid desaturase	68.56	6.00E-139	Resistance to drought and low temperature	Synthesis of fatty acids of the membrane	(Torres-Franklin *et al*., 2009)
scaffold_3_pilon: 8244802	DEAD-box ATP-dependent RNA helicase 5	90.91	4.00E-61	Tolerance to drought, salt and cold stress in wheat	Plant growth, development and abiotic stress	(Ru *et al*., 2021)
scaffold_18_pilon: 5863225	Probable fructokinase-6, chloroplastic	91.18	5.00E-44	–	Fructose phosphorylation	(Stein and Granot, 2018)

OC, pulp oil content; LL, leaf length; LN, number of leaves; EFM, endocarp fresh mass; HDM, husk dry mass; FM, total fruit mass; PFM, pulp fresh mass; BW, bunch weight. Full locus annotations are provided in Supplementary Information 2.

#### Structure under selection

**Fig. 4.**
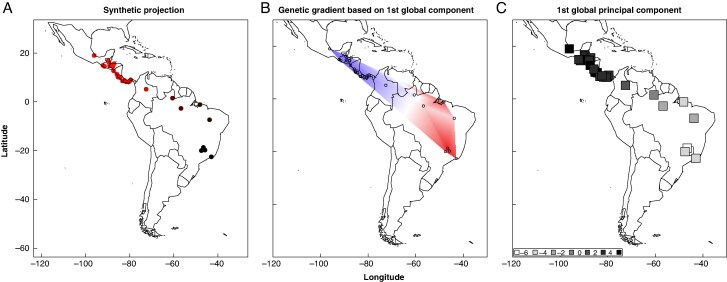
Spatial principal component analysis (sPCA) based on 164 outlier SNPs for 70 samples of *Acrocomia aculeata*. Based on the significance from the Monte Carlo test, only the first global component is shown. (A) Synthetic projections summarizing the representation of values across all significant principal components using an RGB colour gradient. (B) Genetic gradient based on the first global component, with shades of colour indicating close genetic relationships. (C) Significant component represented independently using squares in greyscale, where the intensity of grey reflects the sample’s position on the specific component.

**Fig. 5.**
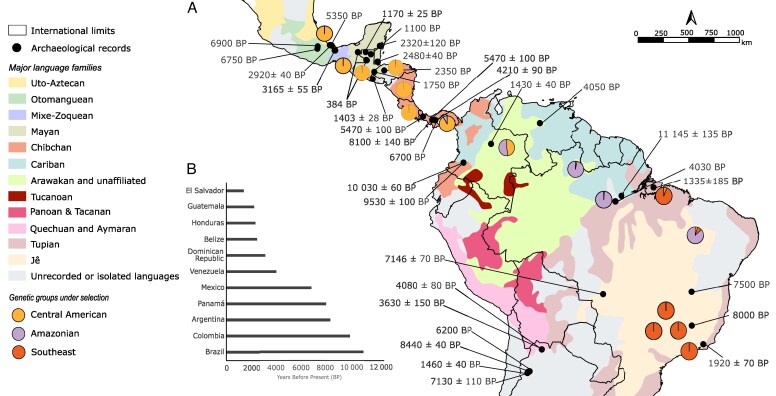
Population structure based on 164 outlier SNPs from 70 samples of *Acrocomia aculeata* and review of the spices’ archeological records in the Neotropics (A) Pie charts showing the average sNMF ancestry coefficients for each genetic group represented by different colours. Distributions of the major language families of the New World at 1500 BP (Bellwood, 2024), and the archaeological records of *A. aculeata* (in years before present, BP). (B) Summary of the earliest archaeological records of *A. aculeata*. A complete database of records is available in Supplementary Material S6.

Using only outlier SNPs, all four methods (sNMF, DAPC, NJ and sPCA) consistently suggested three genetic groups: Central America, Amazon region and southeastern Brazil (Figs 4 and 5; Supplementary Data Figs S9 and S10). This regional division is strongly supported by the sPCA, in which only the global component showed significant relevance (Table S4; Fig. S11), indicating a clear genetic gradient among populations from Central America to southeastern Brazil. Interestingly, the Pará populations again clustered with southeastern Brazil rather than with the Amazon region. Similarly, the Minas Gerais population showed some differentiation from the rest of the Southeastern group (Fig. 4C), although this local structure was not statistically significant. The Mantel test suggested a strong and significant correlation between genetic and geographical distance (*r* = 0.71, *P* < 0.0001; Fig. S12; Table S7), supporting the influence of isolation by distance. The same patterns of population structure were observed in the pairwise *F*_ST_ estimated (Fig. 2B). This elevated differentiation supports the presence of region-specific selective pressures and suggests limited gene flow between groups.

#### Archaeobotanical remains

In total, we identified 58 archaeobotanical records of *A. aculeata* from 11 countries (Fig. 5A). Of these, 66 % were charred endocarp remains, 10 % were artefacts made from *A. aculeata* fibres (e.g. cords), 10 % were seeds, 7 % pollen and 3 % phytoliths. Regarding their context, over 75 % of the records were associated with food preparation or consumption, 12 % with tool or material production, and 9 % with ceremonial use. The oldest records were from South America, in Monte Alegre (Pará, Brazil) and San Isidro (Colombia). In Central America, the earliest records come from the Veracruz region in Mexico (Fig. 5B). In both cases, these early findings correspond to the locations of the first Palaeoindian occupations in each region. Compared with the major language families and the genetic groups, 33 % of the records within the Amazon group were from the Jê major language family, 33 % from the Cariban family, 33 % from the Chibchan family and 1 % from the Arawak family. For the Southeast group 80 % of the records were from the Jê major language family and 20 % from Tupian. For the Central America group, 28 % were from Chibchan family, 44 % from the Mayan family and 28 % from the Otomanguean family.

#### Potential niche modelling analysis during the Pleistocene

**Fig. 6.**
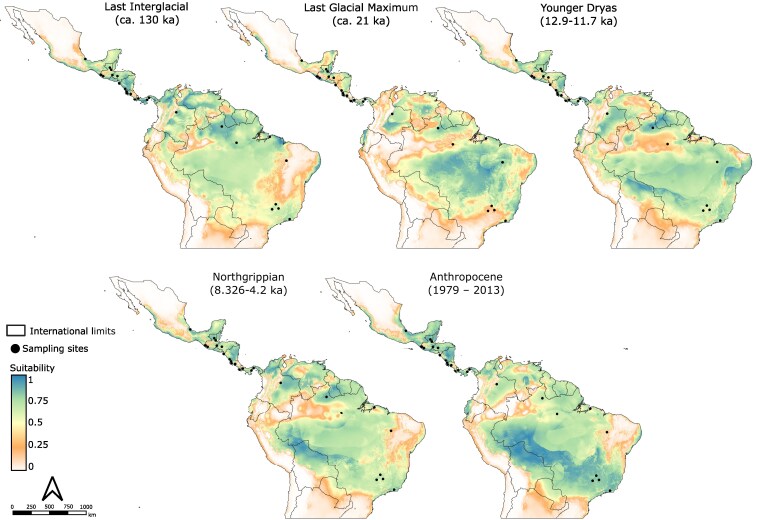
Potential distribution maps of *Acrocomia aculeata* during the late Pleistocene–early Holocene. Values of potential suitability vary from 0 (beige/white) to 1 (blue). The modelling is based on 1303 occurrence records and represents the mean prediction across four different algorithms. Black points represent the sampling locations used in this study. Past bioclimatic variables were obtained from the PaleoClim database (http://www.paleoclim.org).

In total 1303 unique occurrences were used for ecological niche modelling. The AUC (0.947–0.993) and TSS values (0.818–0.941) indicated good model performance and overall consistency (Supplementary Data Table S6). The models suggested significant shifts in environmental suitability and distribution ranges for *A. aculeata* across the late Pleistocene to early Holocene (Fig. 6). In Central America, the most notable changes occurred in the Veracruz region (Mexico) and the Nicaraguan Depression. In South America, major shifts were observed in savanna regions such as the Orinoco Basin (Colombia/Venezuela) and the Brazilian Cerrado, while the Brazilian Amazon region showed relatively stable suitability. Overall, the greatest contraction in suitable habitat occurred between the Last Interglacial and the Last Glacial Maximum, followed by expansion and increased suitability from the Younger Dryas to the present.

## DISCUSSION

The evolutionary history of *Acrocomia* remains partially unresolved, but phylogenetic evidence suggests a divergence approximately 34.99–33.04 Mya (Baker and Couvreur, 2013; Meerow *et al*., 2015). This is supported by the oldest fossil record attributed to the genus, dated to the Middle Oligocene (Dransfield *et al*., 2008). These findings suggest a South American origin for the genus, with expansion and diversification beginning during the Oligocene–Miocene transition and driven by glacial–interglacial climatic oscillations throughout the Cenozoic (Dransfield *et al*., 2008; Baker and Couvreur, 2013). Because phylogenetic turnover in Neotropical palms is shaped largely by dispersal barriers and niche evolution driven by environmental factors (Eiserhardt *et al*., 2013), in the following sections, we examine the geological, climatic and human-mediated processes that have shaped the evolutionary trajectory of *A. aculeata* and *A. totai*, providing context for their current genetic structure and diversity patterns.

### Pre-human configuration

Dispersal barriers can be geological or less evident environmental constraints (Eiserhardt *et al*., 2013). This appears to be the case for South America. The Roraima group is situated within the savannas of Guyana (Amazonian savannas ‘*Lavrado*’) a unique ecosystem rich in endemism (Borghetti *et al*., 2023). Although this ecosystem is currently isolated, the sNMF results suggest a possible historical connection with Colombia (Fig. 1). During the Neogene, the Colombian Llanos, the Gran Sabana in Venezuela and northern Roraima formed a continuous belt of tropical forest, which underwent a process of savannization during the Pleistocene, becoming savanna islands surrounded by tropical forest (Rull *et al*., 2015). While some Pleistocene refugia hypotheses propose connections between Amazonian savannas and other ecosystems (Hoorn *et al*., 2023), geological, genomic and ecological niche modelling reject this scenario. Instead they support long-term stability and independent evolution of Brazilian savannas, probably starting 130 kya (Ratter *et al*., 2003; Buzatti *et al*., 2018; Resende-Moreira *et al*., 2019; Hoorn *et al*., 2023; Kern *et al*., 2023). Our models show no Quaternary shifts in *A. aculeata* suitability for the Roraima region (Fig. 6), and the Mantel test suggests strong isolation by distance in our populations (Supplementary Data Fig. S12), reinforcing the idea of its isolation. This explains Roraima’s high *F*_ST_, elevated inbreeding and low heterozygosity (Fig. 2A; Table 1).

The Maranhão and Mineiro groups occur within the Brazilian Cerrado, the largest Neotropical savanna. Most of this biome diversified within the last 10 Myr, with most lineages emerging in the past 4 Myr, strongly influenced by Quaternary climate oscillations (Lohmann *et al*., 2013; Thode *et al*., 2019; Borghetti *et al*., 2023). As a result, the Cerrado is divided into distinct phytogeographical provinces (Ratter *et al*., 2003), including the north-eastern Cerrado (NC), where the Maranhão groups occur. During the Quaternary, many NC plant species expanded their range, probably facilitating past connections with Amazonia (Bueno *et al*., 2017; Buzatti *et al*., 2017, 2018; Vieira *et al*., 2019; Pantoja *et al*., 2025), which may explain the moderate differentiation between the Amazonas and Maranhão groups, and their clustering in the DAPC, as a relatively recent connection (Supplementary Data Fig. S1). Interestingly, Maranhão had the highest observed heterozygosity and was the only group with a negative inbreeding coefficient (Table 1). This unusual pattern may reflect ongoing or historical gene flow with Amazonas populations, contributing to the maintenance of elevated genetic diversity. Nevertheless, further studies are needed to test this hypothesis and to provide a deeper understanding of admixture dynamics in these populations.

The Mineiro group occurs in the Central Cerrado (CC), while the Southeast group included samples from São Paulo (Southern Cerrado, SC), Rio de Janeiro (Atlantic Forest) and Pará (Amazon Forest). The clustering of SC–Atlantic Forest may reflect Cerrado expansion during the Last Interglacial, which might have facilitated gene flow between ecosystems (Bueno *et al*., 2017; Vieira *et al*., 2019). This was followed by contraction during the Last Glacial Maximum, when CC served as stable Quaternary refugia, and later contributed to the recolonization of other Cerrado areas (Bueno *et al*., 2017; Buzatti *et al*., 2017). This pattern has been observed in multiple plant groups (Lohmann *et al*., 2013; Bueno *et al*., 2017; Buzatti *et al*., 2017; Resende-Moreira *et al*., 2019; Thode *et al*., 2019), including *Acrocomia* (Pantoja *et al*., 2025). This may explain the higher genetic diversity of the CC population compared with other areas (Table 1), and the admixture of some individuals from Rio de Janeiro with Minas Gerais (Fig. 1). The genetic divergence between the Southeast and Mineiro groups may reflect the occupation of distinct refugia during the Pleistocene (Fig. 6).

The most surprising result is the consistent clustering of Belém do Pará (Amazonia) and São Paulo (SC) across all analyses, using both neutral and selected SNPs (Figs 1 and 2C; Supplementary Data Fig. S3). Similar results have been observed in previous studies in *A. aculeata* (Díaz-Hernández *et al*., 2021). One possible explanation could be dispersal through cattle ranching. The fruits of *A. aculeata* are consumed by cattle, which are considered one of the main dispersal agents (Scariot, 1998; Viera *et al*., 2016), significantly contributing to the maintenance of high recruitment rates by regurgitating or excreting the endocarps containing the seeds along with their faeces (Viera *et al*., 2016). Given the expansion of cattle ranching in Brazil after the colonial period (Larrea-Alcázar *et al*., 2021), it is plausible that the fruits were dispersed over long distances, which could explain the clustering of individuals from the northern region with those from other regions. Another possibility is human influence, as early Amerindian populations actively contributed to the dispersal of *A. aculeata* (discussed in the next section).

*Acrocomia totai* formed a distinct and well-supported genetic cluster, consistent with its classification as a separate species from *A. aculeata* (Díaz-Hernández *et al*., 2021; Vianna and Campos-Rocha, 2025). Although the divergence time between *Acrocomia* species remains unknown, the environmental differences in their distribution ranges are likely to have driven genomic differentiation (Pantoja *et al*., 2025). This is further supported by morphological distinctions, including fruit biometry, leaf anatomy and floral structure (Meyer *et al*., 2024). In addition, *A. totai* exhibited higher levels of genetic diversity and a greater number of private alleles (Table 1), potentially reflecting a more ancient diversification, a stable demographic history and greater historical population connectivity across its range (Pantoja *et al*., 2025). Nonetheless, to fully characterize the genetic landscape of *A. totai*, further studies including individuals from the entire geographical distribution of the species are needed.

For the Central American group, Morales-Marroquin *et al*. (2025) identified the Nicaraguan Depression and the Talamanca Mountains systems as key biogeographical barriers that limit gene flow between natural populations of *A. aculeata* into the observed three genomic clusters. They proposed two colonization routes for the region: an early migration from South America to the Maya Block (Northern Central America), and a more recent recolonization of lower Central America following the submersion and re-emergence of the Panama Isthmus. The Mesoamerican group, linked to the first migration during the late Eocene–Oligocene (Baker and Couvreur, 2013; Antonelli *et al*., 2018; Cano *et al*., 2022), exhibits the highest number of private alleles, along with low within-group differentiation (Fig. 2A; Table 1). These metrics are consistent with expectations for an older, stable population that retained ancestral diversity. In contrast, Panamanian group shows reduced diversity and higher differentiation, aligning with a scenario of founder effects during late Miocene–Pliocene dispersal (10–3.5 Mya) (Gutiérrez-García and Vázquez-Domínguez, 2013; Bacon *et al*., 2015). During the Holocene, the Orinoco savanna (Colombia) was used and transformed by groups migrating along the so-called Atlantic Route from the eastern Isthmus of Panama (Rull *et al*., 2015). This could explain the admixture patterns between Panama and Colombia revealed by sNMF and sPCA, along with niche modelling, suggesting that northwest South America populations (probably Orinoco Llanos region) and Indigenous peoples played a significant role in this secondary recolonization wave.

### Outlier SNPs and the putative role of human selection in *Acrocomia aculeata*

The population genetic structure revealed by the analysis of outlier SNPs revealed two *A. aculeata* clusters in South America: one in the Amazon and another in the Southeast (Fig. 5; Supplementary Data Figs S9 and S10). The oldest archaeobotanical records from the Amazonian region (Fig. 5B), aligned with early Palaeoindian presence (Roosevelt *et al*., 1996; Iriarte *et al*., 2020), highlighting the importance of palms for local subsistence (Balée, 1988; Cámara-Leret *et al*., 2019; Shock and Moraes, 2019; Robinson *et al*., 2021). This is especially evident in research on Amazonian Dark Earths (*Terra Preta*), where palms consistently show a hyperdominance (Clement *et al*., 2015; Levis *et al*., 2017; Iriarte *et al*., 2020). Although *A. aculeata* was not dominant (Roosevelt *et al*., 1996; Watling *et al*., 2018; Robinson *et al*., 2021), it appears as a complementary food source in several sites, particularly in central Amazonia (Balée, 1988; Maezumi *et al*., 2018) and Marajó Island (Balée, 2013). One remarkable case in San Isidro, Colombia, where neither the species’ current distribution nor our models support its natural occurrence in the past (Fig. 6), suggesting human-mediated dispersal beyond its natural range (Morcote-Ríos and Bernal, 2001).

Early human occupations in Southeastern Brazil, appear to have been continuous, with most domesticated plants consumed originating from the Amazon, suggesting relatively recent introductions by human migration (Shock *et al*., 2013). This pattern may also apply to *A. aculeata*, as individuals from Maranhão share genomic ancestry with both the Amazonian and Southeastern clusters (Fig. 5). Interestingly, this region is associated with the origin of the Macro-Jê linguistic family and their subsequent southward migration (Ramallo *et al*., 2013), which could suggest that *A. aculeata* was carried along these migration routes into other regions of South America. This is reinforced by archaeological evidence from Minas Gerais, suggesting continuity in palm use, including *A. aculeata*, although most remains have not been identified to the species level (Isnardis and Linke, 2021; Shock, 2010). This is similar to coastal shellmounds (*sambaquis*) from Rio de Janeiro, where palms were important not only as food sources but also in ritual contexts to the fisher–gardener communities (Scheel-Ybert and Boyadjian, 2020).

It is important to highlight that modern ethnobotanical records from southeastern and southern South America reference the use of both *A. aculeata* and *A. totai*. For example, records from the Xetá communities in Serra dos Dourados (Paraná, Tupian family, Fig. 5) and the Aché people in Paraguay (Tupian–Guaraní family) describe the consumption of protein-rich kernels and the production of flour from the palm heart, the only plant-based flour documented in their diets (Fernandes, 1959; Kozak *et al*., 1979 ; Balée, 2013). However, based on current species distribution, these records are more likely to be *A. totai*. For *A. totai*, its ‘anomalous occurrence’ in the Curuguaty region of Paraguay has been linked to pre-Columbian agriculture (Markley, 1956; Balée, 2013), and the Guaraní people in Argentina use the palm’s leaves for basketry, fishing lines and sewing thread, although the species is also perceived as a malignant tree associated with misfortune (Escalada *et al*., 2023). These examples underscore the need to further investigate the domestication history of *A. totai*, especially in regions where both species, and potentially hybrids, may have been traditionally used.

At the genomic level, several genes under selection in South America can be related to local adaptation to climate, soil composition or ecological interactions (Morales-Marroquín *et al*., 2025) while others may be related to these historical uses of the plant (Table 2). The GDSL esterase/lipase gene has been significantly associated with oil content in *Elaeis guineensis*, where it was predominantly expressed in the mesocarp during fruit development (Zhang *et al*., 2018). In *A. aculeata*, this gene has also been linked to total fruit mass (Couto *et al*., 2024), and a similar role in fruit development has been proposed. The same study associated pulp fresh mass with an extracellular ribonuclease. Ribonuclease E/G-like protein identified in South American groups is localized to chloroplasts where they process primary transcripts from chloroplast operons (Walter *et al*., 2010). Malfunction of this protein is related to reduced photosynthetic activity and retarded growth, and also related to seedling lethality (Walter *et al*., 2010; Fang *et al*., 2025). One last interesting gene is the trihelix transcription factor GT-3b, better known as ZmGT-3b. As part of the trihelix transcription factor (TF) family, it was found to be responsible for coordinating the metabolism of maize for a better growth–defence trade-off (Zhang *et al*., 2021). Other members of the trihelix TF family, such as ZmSh1 and Sh4, are involved in plant growth and development and are associated with the domestication syndrome of non-shattering in rice and maize (Alam and Purugganan, 2024), an important agronomic trait in those plants.

Central America encompasses two major cultural areas, Mesoamerica and Lower Central America (Geurds, 2018), where *A. aculeata* has been part of the traditional Milpa agroecosystem since the Archaic period (Lentz, 1990; Ford *et al*., 2023; Morell-Hart *et al*., 2023). The oldest records derive from the Chibcha–Chocó region (Chibcha family, Fig. 5), during the Early Preceramic period (ca. 9000–7400 BP; Ranere and Cooke, 2021), where its abundance suggests intensive harvesting to supplement sugars and oils in the diet (Cooke, 1992; Dickau, 2010; Geurds, 2018). Its widespread presence in both western and central Panama has been interpreted as evidence of regional significance, ‘perhaps the most important’ (Cooke and Ranere, 1992). Smith (1980) proposed its introduction as an allochthonous domesticated species and suggested human selection for larger fruits over time based on archaeological records, although its domestication status was never fully determined.

In Mesoamerica, its presence at Coxcatlán Cave (Tehuacán Valley) during the Archaic is evidence of consumption and human-mediated dispersion and maintenance (Smith, 1967), as supported also by our modelling (Fig. 6). Subsequent records for the Olmec Formative period (5000–3000 BP; Otomanguean family; Fig. 5) support its role for food, medicine and possibly wine (VanDerwarker, 2006; VanDerwarker and Kruger, 2012). The Olmecs may have managed it in both infields and outfields near dwellings (see VanDerwarker, 2006, fig. 4.16, p. 112). Most records derive from the Maya (Formative period onward; Mayan family; Fig. 5.), where it was one of ∼160 useful tree species maintained in Milpa systems for economic and symbolic purposes (Lentz, 1990; Morell-Hart *et al*., 2023), with evidence of active cultivation in some areas (Rushton *et al*., 2013; Morales-Marroquin *et al*., 2025). Beyond dietary uses, flowers were also found in ceremonial tombs, probably valued for the symbolic meaning of their yellow colour (McNeil, 2012).

One of the most lasting uses in Central America is the production of *vino de coyo* (wine) for at least the last 6000 years (VanDerwarker and Kruger, 2012). The earliest written records appear in the biography of Christopher Columbus which describes the Indigenous people of Veragua (Panamá City) making ‘wine from trees that resemble palms (…), smooth like other trees, but with spines on the trunk as long as those of a porcupine. From the pith of these palms, pressing and squeezing it, they extract the juice to make wine (…), which they consider very good and highly valued’ (Colón, 1947). The beverage was also recorded in the 16th century as a dietary supplement during periods of scarcity (Terán and Heilskov Rasmussen, 1994), a practice that is present today in rural communities across Mexico, known as ‘Taberna’ (Ambrocio-Ríos *et al*., 2021), Honduras, known as ‘Mocorón’ (Balick, 1990), and Costa Rica (Sylvester *et al*., 2012), where the production of *vino de coyol* continues to support local economies and cultural identity. Additionally, the fruit is used for making sweets in Mexico and Guatemala, and as an ornamental plant in Panama (Morales-Marroquin *et al*., 2025).

The Central American group also had many outlier SNPs in genes possibly related to these historical uses (Table 2). Some outlier SNPs were in zinc finger proteins (ZFPs), which were associated with oil content and leaf traits in *A. aculeata* (Couto *et al*., 2024), as well as to bunch weight in *Elaeis oleifera × Elaeis guineensis* hybrids (Osorio-Guarín *et al*., 2019). The TF zinc finger protein nutcracker (ZFP-NUT) regulates responses to abiotic stress, particularly influencing roots (Long *et al*., 2015; Ling *et al*., 2023) and photoperiodic flowering through sugar transport and metabolism, processes that may influence oil production (Seo *et al*., 2011). The importance of processes related to metal ion binding has also been identified in other studies (Couto *et al*., 2024). Another example is the putative disease resistance protein RGA3, associated with leaf spot resistance in *E. guineensis* (Wibowo *et al*., 2023) and commonly involved in foliar disease resistance across various crops (Sekhwal *et al*., 2015).

One of the most distinctive features of Central American *A. aculeata* populations is the presence of adult dwarf phenotypes (Morales-Marroquin *et al*., 2025). We found outlier SNPs in two genes potentially involved with dwarfism: reduced wall acetylation 3 (RWA) and ABC transporter B family member 20 (ABCB). RWA genes are involved in cell wall polysaccharide acetylation, increasing cell wall digestibility and facilitating oil extraction. In *Arabidopsis*, a single RWA mutation reduced cell wall acetylation by ∼20 % without phenotypic effects, while quadruple mutants showed a 63 % reduction and severe dwarfism (Manabe *et al*., 2013). Notably, genes encoding RWA proteins were positively correlated with oil production traits in *A. aculeata*, such as endocarp fresh mass and husk dry mass (Couto *et al*., 2024). ABCB transporters are associated with plant architecture: mutations in these genes confer dwarf phenotypes in maize (*br2*), sorghum (*dr3*) and watermelon (*Cldw-1*), without compromising yield (Banasiak and Jasiński, 2022). Dwarfism is a valuable agronomic trait, central to the agronomic success in several crops and desirable in modern fruit and oilseed production (Warang and Gawas, 2025; Bhujbal *et al*., 2025), so this phenotype was probably subject to selection by Mesoamerican people.

### Independent domestication trajectories in Central and South America

Domestication and management strategies often depend on the specific plant resource being exploited (Huang *et al*., 2022), and Indigenous and traditional societies across the Americas appear to have applied this principle to *A. aculeata* through what seems to be a process of independent domestication. This pattern is not unique to *A. aculeata*, but rather reflects a broader trend observed in several Neotropical crops such as *Phaseolus* sp., *Cucurbita* sp., *Chenopodium* sp. (Pickersgill, 2007) and *Zea mays* (Costa *et al*., 2024), as well as tree species such as *Theobroma cacao* (Lanaud *et al*., 2024) and the only fully domesticated Neotropical palm *Bactris gasipaes* (Clement *et al*., 2017).

In South America, the available evidence suggests that *A. aculeta* was used primarily for fibre production and oil extraction within predominantly extractivist systems (Solidario De Souza Benatti *et al*., 2025). This is consistent with the outlier SNPs in genes associated with lipid metabolism and cell wall modification. It is important to note that documented records from this region are still scarce (Iriarte *et al*., 2020; Peripato *et al*., 2023). Recent studies emphasize that large areas of anthropogenic influence in Amazonia remain unexplored and may reveal greater abundance of human-associated species such as *A. aculeata* (Peripato *et al*., 2023). In contrast, Central America presents more extensive and diverse records, including of cultivated and protected populations (Lentz, 1990; Cooke and Ranere, 1992; VanDerwarker, 2006; Rushton *et al*., 2013). In this region, *A. aculeata* was widely used for its sugar content, particularly in fermented beverages, and was probably selected for traits such as larger fruits (Smith, 1967), which may have favoured different human selection and dispersal (Lentz, 1990). This highlights the need for greater academic attention to this region (Morales-Marroquín *et al*., 2022).

### Opportunities for just energy transition in *Acrocomia*

In recent years, the concept of a Just Energy Transition (JET) calls for a shift to low-carbon energy sources based on a systemic transformation that addresses historical social inequalities and avoids exacerbating existing vulnerabilities (Rios-Ocampo *et al*., 2025). Under a JET perspective, *Acrocomia* holds promise not only as a renewable and decarbonized energy source, but also as a driver of inclusive socioeconomic development. Its cultivation and valorization could contribute to safeguarding equity and welfare, particularly for the small-scale producers and traditional communities that have historically managed and used the species (García-García *et al*., 2020; Vargas-Carpintero *et al*., 2022; Solidario De Souza Benatti *et al*., 2025). Our findings demonstrate that the genetic diversity of *A. aculeata* and *A. totai* has long been shaped by multiple Latin American people, emphasizing that an energy transition centred on *Aacrocomia* must also be a just transition, ensuring true sustainability through the inclusion of the people and knowledge systems that have contributed to its domestication across history.

## Supplementary Material

mcaf282_Supplementary_Data

## Data Availability

The VCF file containing the final SNP matrix is publicly available in Figshare at: https://doi.org/10.6084/m9.figshare.29228240.v1. The rest of the information is available in the supplementary material.

## SUPPLEMENTARY DATA

Supplementary data are available at *Annals of Botany* online and consist of the following. Figures S1–S15 are provided in the supplementary material. Tables S1–S8 are available in Supplementary Information S1, and the results of the outlier analyses and functional annotation for the three datasets are presented in Supplementary Information S2.

## AI ASSISTANCE

Artificial intelligence was used in the translation of the manuscript.
