# Supplementary material for: From genomics to domestication: biocultural history of the Neotropical palms Acrocomia aculeata and Acrocomia totai
Source: Ann Bot. 2025 Nov 17;137(3):725–43. doi: 10.1093/aob/mcaf282 (PMC13006974; doi:10.1093/aob/mcaf282)
Supplement: mcaf282_Supplementary_Data [file mcaf282_supplementary_data.zip › Sumplementary Material.pdf]

Supporting information

From genomics to domestication: biocultural history of the Neotropical palms *Acrocomia aculeata* and *Acrocomia totai*

Eduardo Antonio Monge-Castro[1]*; Jonathan Morales-Marroquín[2]; Brenda Gabriela Díaz-Hernández[3]; Suelen Alves Vianna[5]; Ana Flávia Francisconi[1]; Caroline Bertocco Garcia[1]; Matheus Scaketti[2]; Flaviane Malaquias Costa[1]; Alessandro Alves-Pereira[1]; Carlos Augusto Colombo[3]; Maria Imaculada Zucchi[124]

[1]Department of Genetics, Luiz de Queiroz College of Agriculture, University of São Paulo, São Paulo, Brazil.
[2]Department of Genetics and Molecular Biology, Institute of Biology, State University of Campinas, São Paulo, Brazil.
[3]Center of Plant Genetic Resources, Agronomic Institute (IAC), Campinas, São Paulo, Brazil.
[4]Paulista Agency of Agribusiness Technology, Centro-Sul Site (APTA), Piracicaba, São Paulo, Brazil
[5]Department of Research & Innovation, Plant Breeding Division, Acelen Renewable Energy, São Paulo, Brazil.

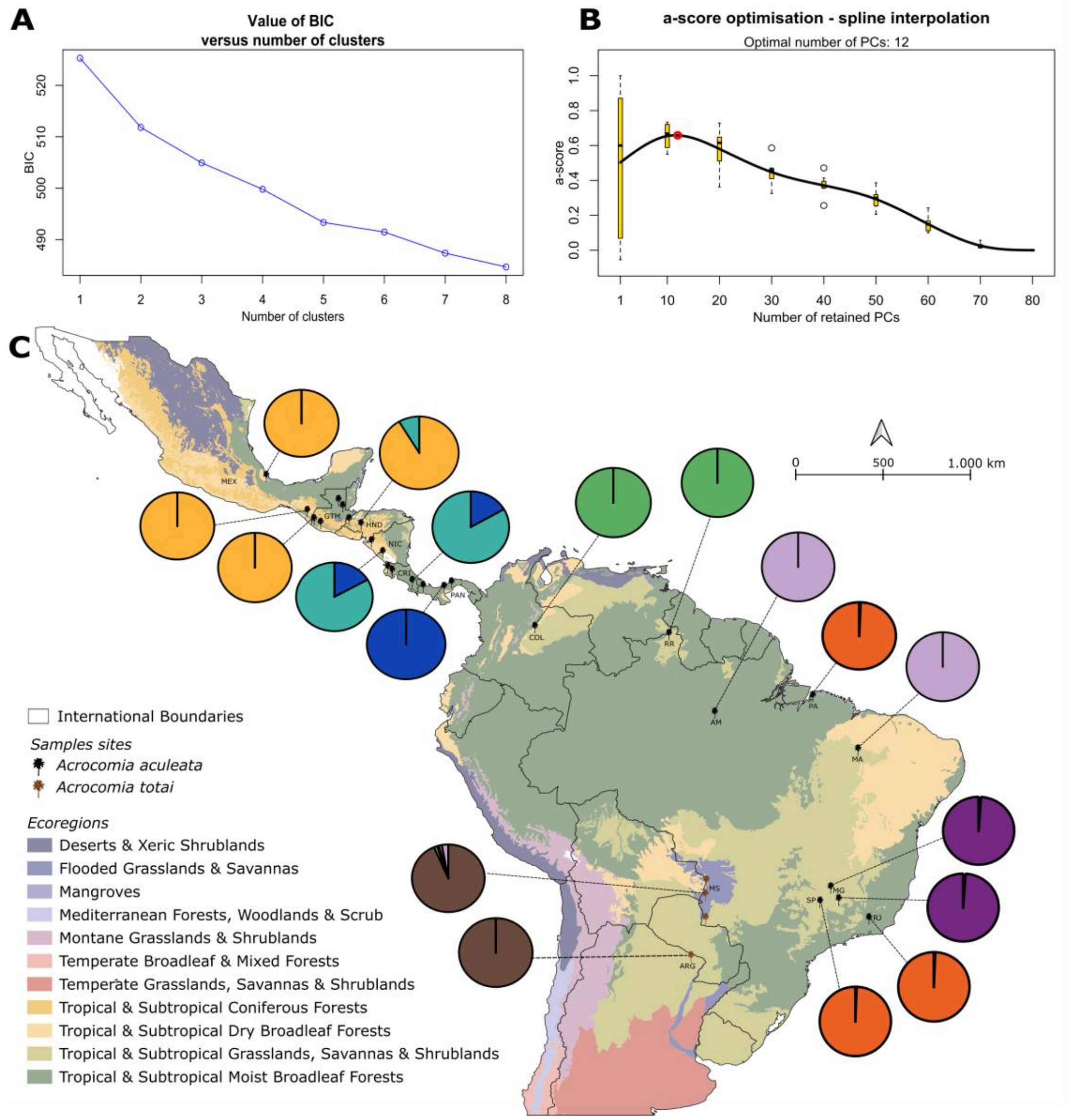

Figure S1. Results from discriminant analysis of principal components (DAPC) based on 4,716 SNPs from 70 samples of *A. aculeata* and 11 of *A. totai*. (A) Number of clusters identified by the k-mean method. (B) Number of PCA retained based on α-score optimization method. (C) Pie charts show the average DAPC coefficients for each genetic group represented by different colors. The different ecoregions are defined according to Dinerstein et al. (2017). Acronyms for countries or states: MEX Mexico, GTM Guatemala, HND Honduras, NIC Nicaragua, CRI Costa Rica, PAN Panama, COL Colombia; and the Brazilian States, MA Maranhão, PA Pará, AM Amazonas, RR Roraima, MG Minas Gerais, SP São Paulo, and RJ Rio de Janeiro.

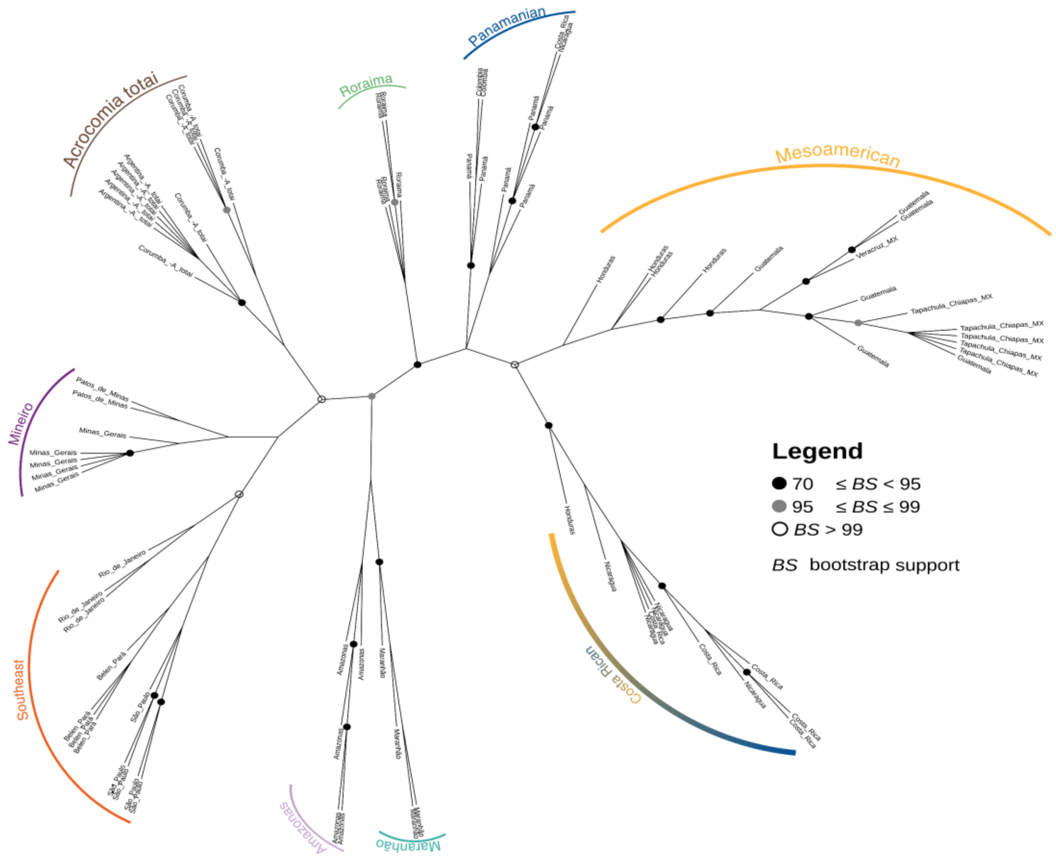

Figure S2. Results from the Neighbor Joining (NJ) clustering method based on Nei's genetic distance based on 4,716 SNPs from 70 samples of *A. aculeata* and 11 of *A. totai*. The dots represent the support value based on 1000 bootstraps. It also shows the genomic groups assigned to each of the cluster identified.

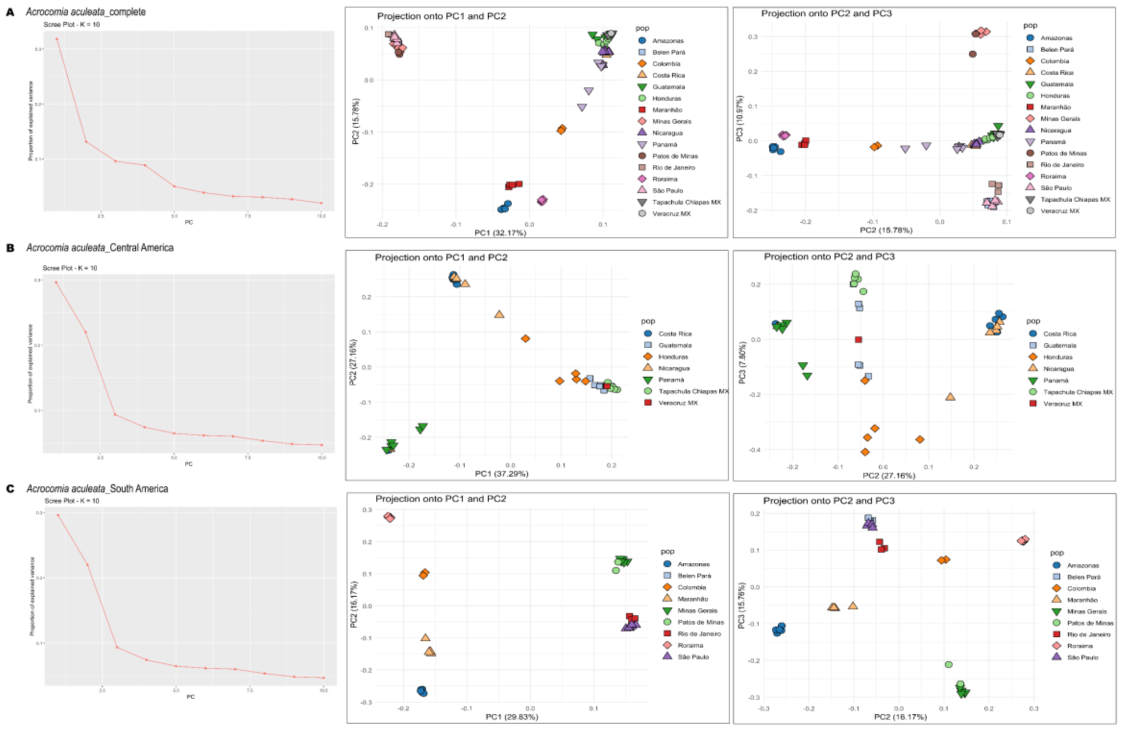

Figure S3. Principal component analysis (PCA) performed with pcadapt for (A) Acrocomia aculeata complete dataset (B) Central America dataset, and (C) South America dataset. The first plot explains the variance retained in each principal component (PC) used for choosing the *K* numbers based on Cattell's rule. The second and third plot shows the first two PCs and the second and third PCs, respectively.

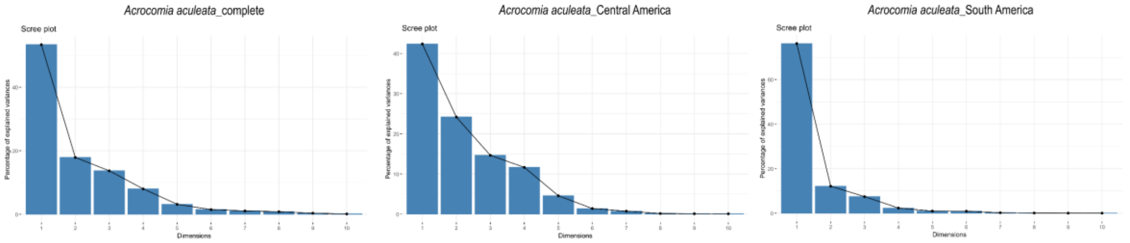

Figure S4. Principal components (PCs) retained that have the higher contribution to the variation determined by the environmental variables. To retain 90% of the observed variance, the first four principal components were retained for the complete and the Central American dataset, while the first three PCs were retained for the South American dataset.

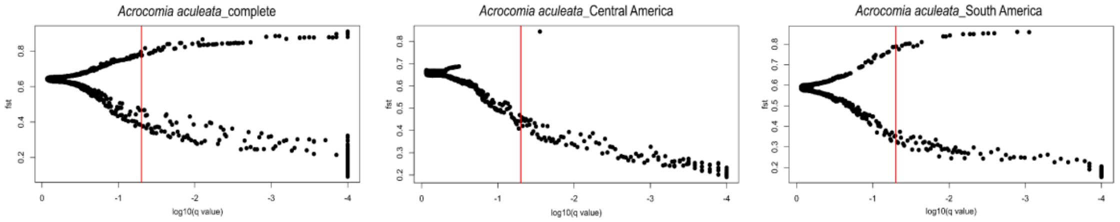

Figure S5. Bayescan plot showing FST estimates against log10(q-values). The vertical red line indicates the cutoff (FDR = 0.05) used for identifying outlier SNPs.
The markers on the right side of the vertical line show all outlier SNP candidates and

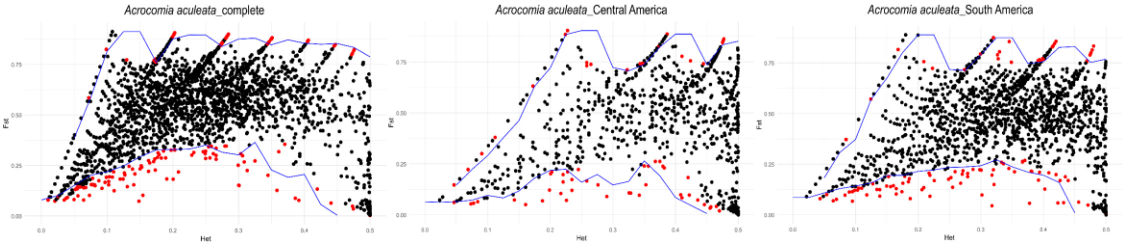

Figure S6. Plot of SNP-specific FST values against expected heterozygosity (*He*) generated using the Fsthet method. The blue lines represent the 95% confidence intervals. SNPs identified as outliers are shown in red.

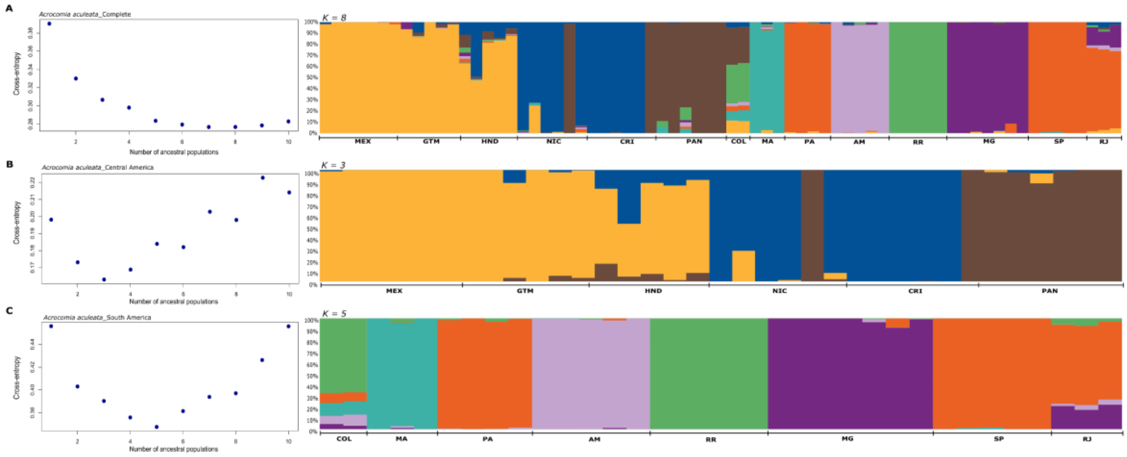

Figure S7. Sparse non-negative matrix factorization (sNMF) performed for outlier detection using Latent Factor Mixed Models (LFMM) for (A) *Acrocomia aculeata* complete dataset (B) Central America dataset, and (C) South America dataset. The first plot explains the cross-entropy estimates for each number of simulated ancestral populations and on the right the bar plots representing the sNMF ancestry coefficients across samples. Acronyms for countries or states: MEX Mexico, GTM Guatemala, HND Honduras, NIC Nicaragua, CRI Costa Rica, PAN Panama, COL Colombia; and the Brazilian States, MA Maranhão, PA Pará, AM Amazonas, RR Roraima, MG Minas Gerais, SP São Paulo, and RJ Rio de Janeiro. Colors are similar as in figure 1.

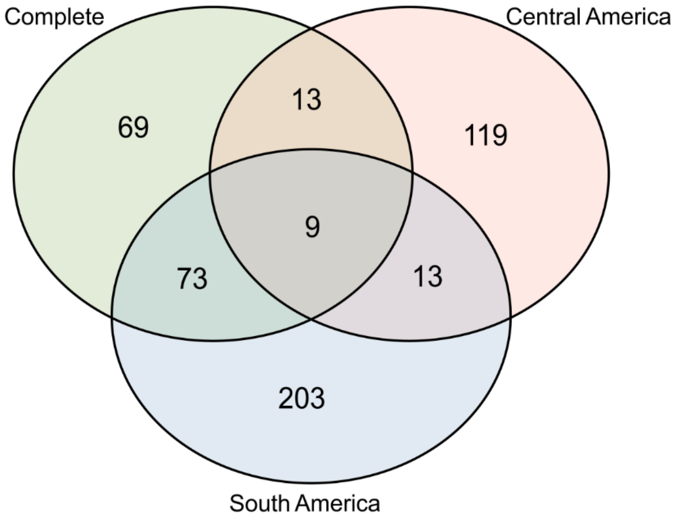

Figure S8. Comparison of the set of outlier SNPs identified for the three datasets.

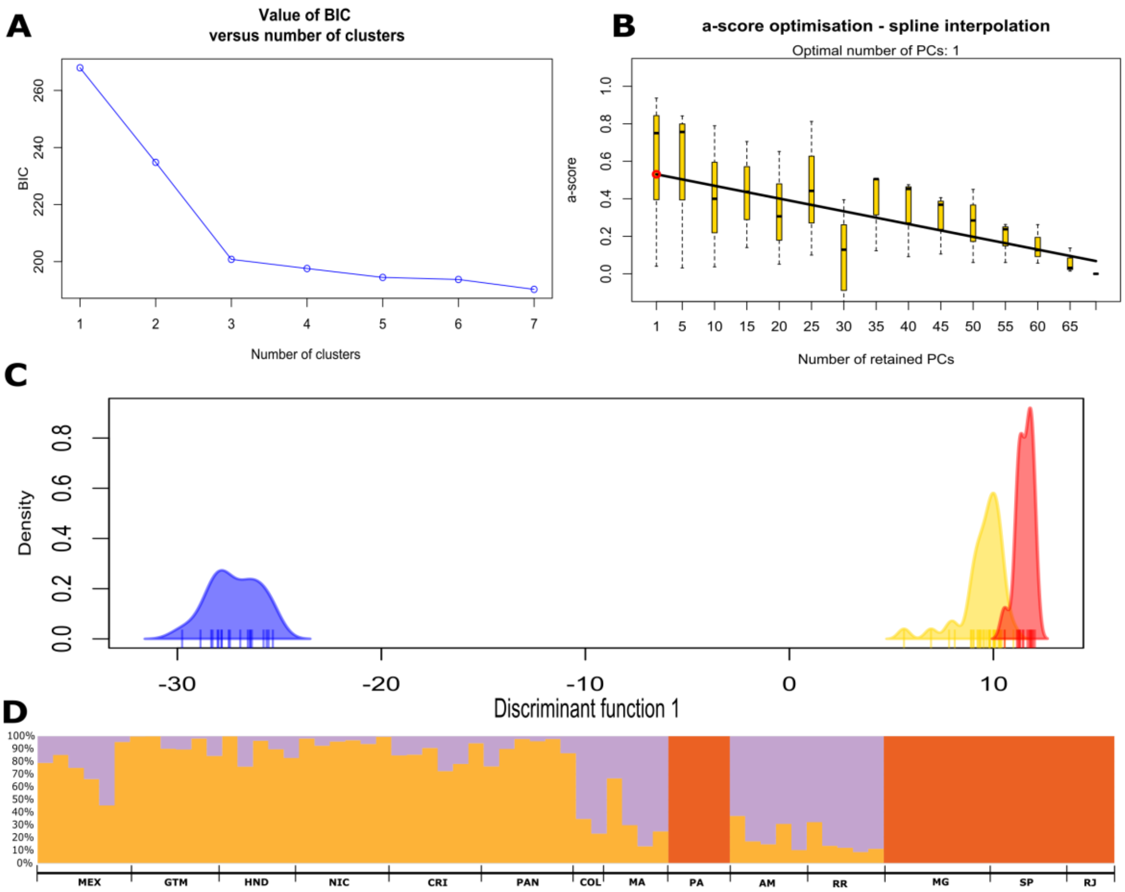

Figure S9. Results from discriminant analysis of principal components (DAPC) based on 164 SNPs outliers from 70 samples of *A. aculeata*. (A) Number of clusters identified by the k-mean method. (B) Number of PCA retained based on α-score optimization method. (C) Scatter plot for each sampling population. Each oval represents the clustering group assigned by the K-means method. (D) Bar plots representing the DAPC coefficients across samples. Acronyms for countries or states: MEX Mexico, GTM Guatemala, HND Honduras, NIC Nicaragua, CRI Costa Rica, PAN Panama, COL Colombia; and the Brazilian States, MA Maranhão, PA Pará, AM Amazonas, RR Roraima, MG Minas Gerais, SP São Paulo, and RJ Rio de Janeiro.

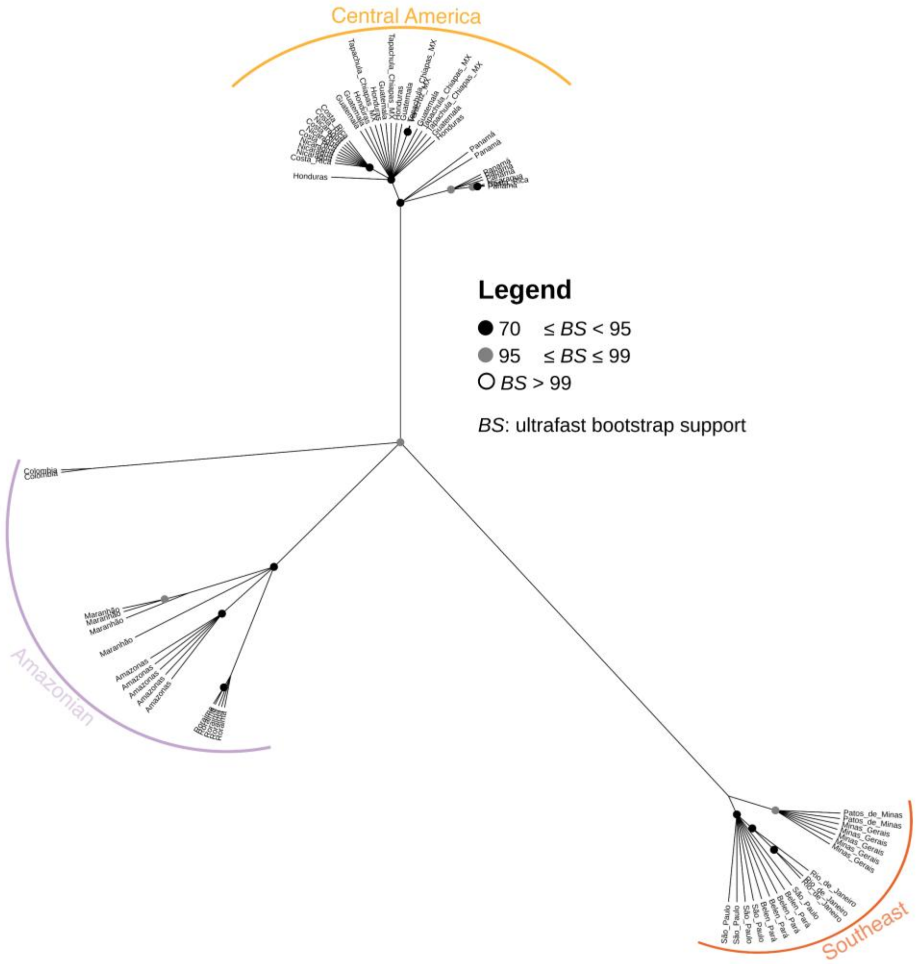

Figure S10. Results from the Neighbor Joining (NJ) clustering method based on Nei's genetic distance based on 164 SNPs outliers from 70 samples of *A. aculeata*. The dots represent the support value based on 1000 bootstraps. It also shows the genomic groups assigned to each of the cluster identified.

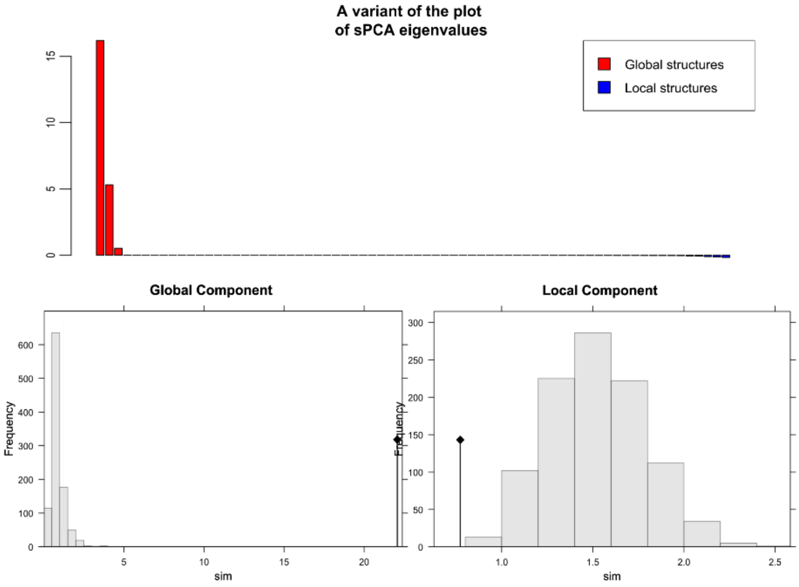

Figure S11. Significance for the global and local structure of the spatial principal component analysis (sPCA) based on 164 SNPs outliers of 70 samples of *Acrocomia aculeata.*

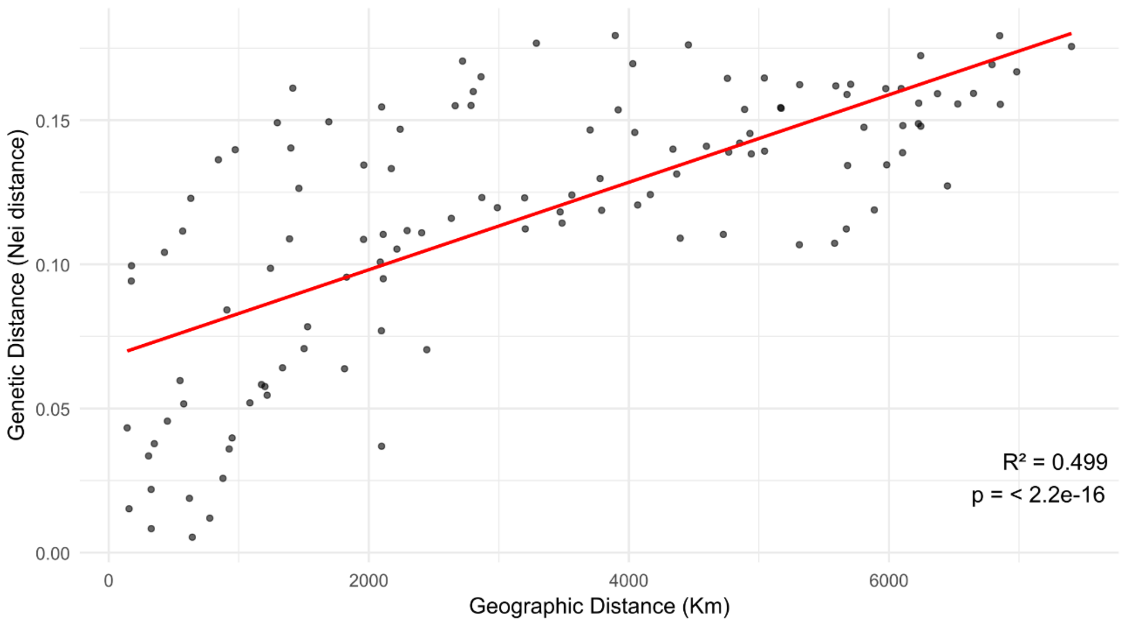

Figure S12. Correlation between genetic and geographic distance based on SNPs under selection. The Mantel test revealed a significant positive correlation between genetic and geographic distances among *A. aculeata* populations ($r = 0.71$, $p < 0.001$), supporting a pattern of isolation by distance.

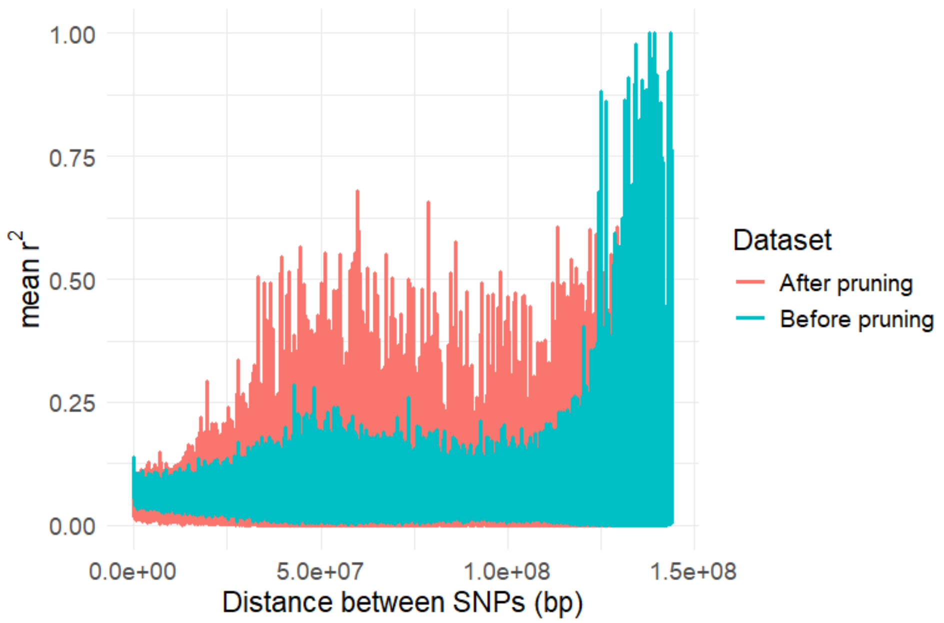

Figure S13. Linkage disequilibrium (LD) decay before and after of pruning. Mean pairwise LD (r^2) is plotted against physical distance (bp) between SNPs. The blue line represents LD calculated from the unpruned dataset (10,955 SNPs), while the red line represents LD after pruning (4,716 SNPs; $r^2 < 0.6$, minimum distance = 150 bp). LD values are generally higher and persist over long distances before pruning, whereas after pruning LD decays more rapidly, with low r^2 values at larger distances.

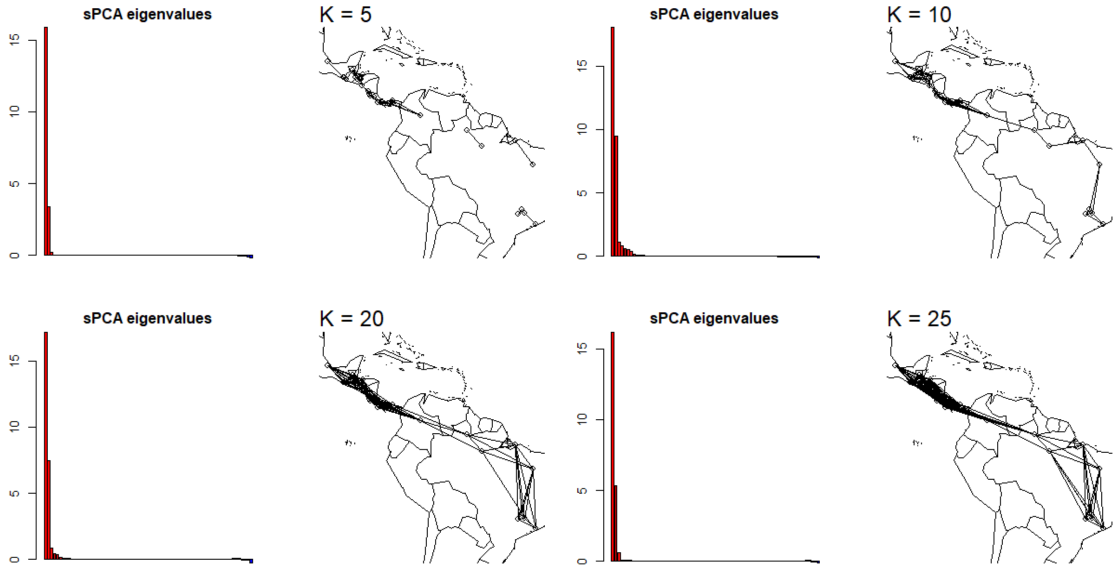

Figure S14. Spatial PCA results across different values of K and thei global and local sPCA eigenvalues for each K. The figure shows the connections among individuals for each K and the relevance of global and local principal components. Among the tested values, K = 20 provides a balanced representation of the spatial structure, capturing connections without overrepresenting them.

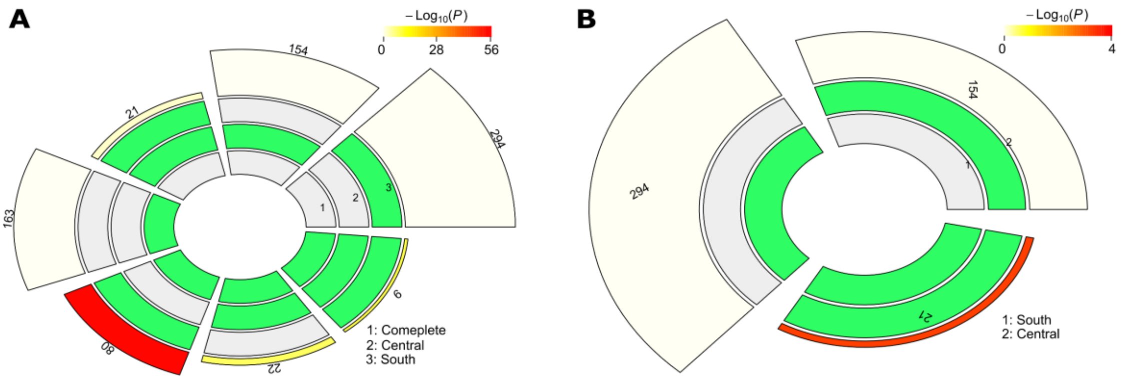

Figure S15. Results of SuperExactTest for outlier SNP overlaps across regions. (A) Overlaps among South, Central, and Complete datasets. (B) Pairwise comparison between South and Central. Numbers indicate observed overlaps, and color scale represents significance (–$\log_{10}$ p-value). Green bands mark the sets included in each intersection.
